# In vivo multiphoton multiparametric 3D quantification of human skin aging on forearm and face

**DOI:** 10.1038/s41598-022-18657-z

**Published:** 2022-09-01

**Authors:** Ana-Maria Pena, Thérèse Baldeweck, Etienne Decencière, Serge Koudoro, Steeve Victorin, Edouard Raynaud, Blandine Ngo, Philippe Bastien, Sébastien Brizion, Emmanuelle Tancrède-Bohin

**Affiliations:** 1 L’Oréal Research and Innovation, 1 Avenue Eugène Schueller, BP22, 93601 Aulnay-sous-Bois, France; 2 MINES ParisTech-PSL Research University, Fontainebleau, France; 3 L’Oréal Research and Innovation, Campus Charles Zviak RIO, 9 rue Pierre Dreyfus, Clichy, France; 4 Service de Dermatologie, Hôpital Saint-Louis, Paris, France

**Keywords:** Ageing, Multiphoton microscopy, Three-dimensional imaging, Translational research

## Abstract

Quantifying skin aging changes and characterizing its 3D structure and function in a non-invasive way is still a challenging area of research, constantly evolving with the development of imaging methods and image analysis tools. In vivo multiphoton imaging offers means to assess skin constituents in 3D, however prior skin aging studies mostly focused on 2D analyses of dermal fibers through their signals’ intensities or densities. In this work, we designed and implemented multiphoton multiparametric 3D quantification tools for in vivo human skin pigmentation and aging characterization. We first demonstrated that despite the limited field of view of the technic, investigation of 2 regions of interest (ROIs) per zone per volunteer is a good compromise in assessing 3D skin constituents in both epidermis and superficial dermis. We then characterized skin aging on different UV exposed areas—ventral and dorsal forearms, face. The three major facts of aging that are epidermal atrophy, the dermal–epidermal junction (DEJ) flattening and dermal elastosis can be non-invasively quantified and compared. Epidermal morphological changes occur late and were only objectified between extreme age groups. Melanin accumulation in suprabasal layers with age and chronic exposure on ventral and dorsal forearms is less known and appears earlier. Superficial dermal aging changes are mainly elastin density increase, with no obvious change in collagen density, reflected by SHGto2PEF ratio and SAAID index decrease and ImbrN index increase on all skin areas. Analysis of the z-dermal distribution of these parameters highlighted the 2nd 20 µm thickness normalized dermal sub-layer, that follows the DEJ shape, as exhibiting the highest aging differences. Moreover, the 3D ImbrN index allows refining the share of photoaging in global aging on face and the 3D SAAID index on forearm, which elastin or fibrillar collagens densities alone do not allow. Photoaging of the temple area evolves as a function of chronic exposure with a more pronounced increase in elastin density, also structurally modified from thin and straight elastic fibers in young volunteers to dense and compact pattern in older ones. More generally, multiphoton multiparametric 3D skin quantification offers rich spatial information of interest in assessing normal human skin condition and its pathological, external environment or product induced changes.

## Introduction

Human skin aging^1^ is a complex phenomenon driven by intrinsic (chronology and genetic) and extrinsic (mainly environmental) factors that progressively modify the skin constituents and impact the global appearance of our skin. One of the most important extrinsic factors is the chronic sun exposure, whose deleterious effects (photo-aging) can easily be seen by comparing the skin areas regularly exposed with the ones protected from sunlight. Clinically, the intrinsic or chronological skin aging results in epidermal and dermal atrophy, skin dryness, reduced skin elasticity, appearance of fine wrinkles, fragility of skin and blood capillaries, whereas the extrinsic aging is characterized by the appearance of pigmented spots, deep wrinkles and telangiectasias. Histologically, the skin aging results in decreased epidermal thickness, even though the number of cell layers remains unchanged, flattening of dermal–epidermal junction, increased mottled pigmentation, dermis atrophy and degenerative dermal changes, most obvious being elastosis in the sun-exposed areas^2^.

Quantifying skin aging changes especially on human face and more generally characterizing its 3D structure and function in a non-invasive way is still a challenging area of research, constantly evolving with the development/improvement of linear and non-linear optical imaging methods and image analysis tools. Methods such as confocal reflectance imaging^3–6^, (line-field confocal) optical coherence tomography^4,7–9^ and multiphoton imaging^10–17^, have paved the way towards a non-invasive live 2D/3D imaging of in vivo human skin, offering means of avoiding performing skin biopsies in several situations and assessing human skin aging in vivo. While all these methods enable morphological characterization of the epidermis, epidermal cells and dermal–epidermal junction with sub-µm resolution, only multiphoton imaging provides specificity for melanin, elastin or fibrillar collagen detection. Its ability to detect intrinsic two-photon excited fluorescence (2PEF) from cellular and extracellular matrix constituents (e.g. keratin, metabolic coenzymes, melanin, and elastin) and second harmonic generation (SHG) signals from fibrillar collagens^18–20^, leveraged by fluorescence lifetime imaging (FLIM) to provide functional information on skin constituents^21^, enabled different applications in dermatological sciences^22–33^.

Although multiphoton imaging enables 3D skin imaging, various ex vivo and in vivo human skin studies focused on assessing dermal aging by computing the mean 2PEF (mainly elastin) and SHG (fibrillar collagens) signal intensities or densities in 2D, at specific fixed depths around 110–160 µm bellow the skin surface^10,11,15,17,34–36^. Based on these data, combination parameters were introduced such as the SHG to autofluorescence aging index of dermis (SAAID) defined as the ratio of the difference to the sum of SHG and 2PEF pixels areas (2D SAAID density-based index)^10^ or pixels intensities (2D SAAID intensity-based index)^11^. The 2D SAAID intensity index generally decreases with age on forearm or gluteal regions^11,15,37,38^ and facial skin cheek area^35^, but a lack of aging variation of this index^34^ or of the mean 2PEF and SHG signal intensities^17^ has also been reported. Its aging decrease can be more pronounced on solar exposed areas characterized by an increased autofluorescence intensity due to elastosis. This index also depends on the gender and the investigated area^6^ making it necessary to select comparable sites when comparing photo-aged to sun-protected skin.

Quantification of an intensity-based SAAID index alone cannot give information on the elastin and collagen fibers densities nor into their respective increase or decrease with aging and one must quantify these parameters individually to correctly interpret the data. Assessing, interpreting, and comparing the dermal fibers intensities results between different studies is quite tricky: one must consider factors such as the excitation power and the attenuation of both the excitation wavelength and of the emitted 2PEF and SHG signals, impacted by variations in epidermal thickness, melanin density and the presence of blood capillaries. For these reasons, density-based parameters^10,12,13,17,38^ could be more robust in assessing the dermal fibers changes as initially proposed by Lin et al*.*^10^. Moreover, integrating the measurements over the whole 3D imaged dermis instead of choosing a fixed z-depth plane could minimize their dependency over factors such as the native skin variability.

Our group, with the development of an automatic 3D epidermal-dermal segmentation^13^, opened the possibility of quantifying human skin in 3D and assessing not only its dermal fibers within the entirely imaged dermis, but also the epidermal and DEJ morphology. We reported for the first time in vivo, the ventral forearm skin aging changes in the 3D parameters of epidermal thickness (decrease), DEJ undulation (flattening), density of elastin (increase) and collagen fibers (no change), and their 3D SAAID density-based index (decrease)^13^. Moreover, to assess the elastin and collagen fibers’ overlapping, we introduced a multimodal 3D organization parameter (normalized imbrication ImbrN)^13^ found to increase with aging on ventral forearm. Recently, by comparison with FLIM bi-exponential and phasor analyses, we have demonstrated how melanin can be detected in 3D by multiphoton Pseudo-FLIM analysis (slope analysis of autofluorescence intensity decays from temporally binned data, compatible with 3D in vivo fast image acquisitions on human subjects)^32,39^. As the epidermal thickness may vary according to anatomical sites, skin ethnicity, aging or products application, we proposed to use both a global 3D epidermal melanin density parameter and a melanin z-epidermal distribution profile allowing to compare skin regions with varying epidermal thickness.

In this work, we propose to take into account all the available 3D rich spatial information offered by multiphoton imaging and perform a global 3D assessment of in vivo human skin aging changes on ventral and dorsal forearms and face temple skin areas, after addressing the question of skin constituent’s variability and robustness of 3D multiphoton parameters for skin assessment.

## Materials and methods

### In vivo human skin—clinical trials

Clinical studies were conducted in Paris, France in accordance with local legal requirements, Principles of the Good Clinical Practices, and the Declaration of Helsinki and all volunteers gave written informed consent. The experimental protocols of studies I and II were approved by the Saint Louis Hospital ethics committee (study I—EC reference 2010/28; study II—EC reference 2008/62). According to French regulations on non-interventional research (Article R1121-2 of August 9th, 2004, version of September 7th, 2006 updated in April 2013), the experimental protocol for study III did not require ethics committee approval.

#### *Study I—skin variability, photo-aging (30–40 vs 55–65 year-old) on ventral forearm—DermaInspect microscope*

(October–November 2010; original data) involved 40 European origin volunteers (20 women and 20 men) in two age groups 30–40 year-old (n = 20) and 55-65 year-old (n = 20) and with skin color determined on the ventral forearm by Individual Typology Angle (ITA)^40^: [41°, 55°] (ITA group “light” skin color, n = 20) and [10°, 28°] (ITA group “tan” skin color, n = 20). Each age and skin color ITA group includes 5 women and 5 men. Colorimetry (Datacolor, Montreuil, France) measurements were performed on ventral forearm. Multiphoton imaging was performed in 4 adjacent ROIs, arbitrarily chosen within the central part of a 0.8 cm^2^ region (same location as for colorimetry).

#### *Study II—photo-aging (18–25 vs 70–75 year-old) on ventral and dorsal forearms—DermaInspect microscope*

(January–February 2009; partially published in^13,32^) involved 15 young (18–25 year-old) and 15 aged (70–75 year-old) European origin female volunteers with Fitzpatrick phototypes I–IV. Multiphoton imaging was performed on ventral (2 adjacent ROIs) and dorsal (1 ROI) forearms (less exposed vs. sun exposed skin areas).

#### *Study III—photo-aging (18–25 vs 70–75* year-old*) on dorsal forearm and face temple area—MPTflex microscope*

(April–May 2016, original data) involved 15 young (18–25 year-old) and 15 aged (70–75 year-old) European origin female volunteers with Fitzpatrick phototypes I to IV and skin color ITA [28°, 55°] (ITA groups “light” and “intermediate”) on the ventral side of the upper arm at screening. Colorimetry (Datacolor, Montreuil, France) measurements were performed on dorsal forearm and face temple area. Multiphoton imaging was performed within 3 adjacent ROIs, on dorsal (mostly exposed) forearm and face temple (exposed) areas, the temple area being chosen in alignment with the internal and external orbital canthus.

For all these studies, during data quality check, we mainly excluded the multiphoton z-stacks with important volunteer movements during acquisition. The final number of volunteers and ROIs for each study is given in “Supplementary information S1”.

### In vivo 3D multiphoton imaging—2PEF-FLIM (4 time channels)/SHG z-stacks

Z-stacks of images were acquired using either DermaInspect® or MPT*flex™* systems (JenLab GmbH, Germany) integrating TCSPC detectors, a FLIM module (SPC-830, Becker & Hickl, Berlin, Germany) and an 80 MHz IR fs pulsed laser (MaiTai and, respectively, MaiTai XF1, Spectra-Physics, Mountain View, CA, USA).

With DermaInspect®, images were acquired upon 760 nm excitation and detection in the 390–650 nm (2PEF signal) and 380 ± 7 nm (SHG signal) wavelength ranges, using a 40x /1.3 NA oil immersion objective (exponentially increased excitation power from 12 mW at the skin surface up to 47 mW at depths exceeding 75 µm), as previously described^13,24,32,41,42^. Image characteristics: 3D z-stacks (70 images; 2.346 µm z-step); 130.3 × 130.3 µm^2^; 511 × 511 pixels (0.255 µm/pixel) × 4 time channels (2.08 ns/time channel), 0–8.33 ns time range, maximum intensity decay at 1.33 ns; 28 µs pixel dwell time; 7.4 s/image; 9.4 min/3D z-stack.

With MPT*flex™*, images were acquired in similar conditions to DermaInspect® except for the following parameters: 2PEF signal detection in the 409–650 nm; exponentially increased excitation power from 10 mW at the skin surface up to 30 mW at depths exceeding 75 µm; 2.54 µm z-step; 204,4 × 204,4 µm^2^; 511 × 511 pixels (0.4 µm/pixel); 16.8 µs pixel dwell time; 4.4 s/image; 5.2 min /3D z-stack of 70 images.

### In vivo 3D automatic skin layers segmentation and constituents’ quantification

**Figure 1:**
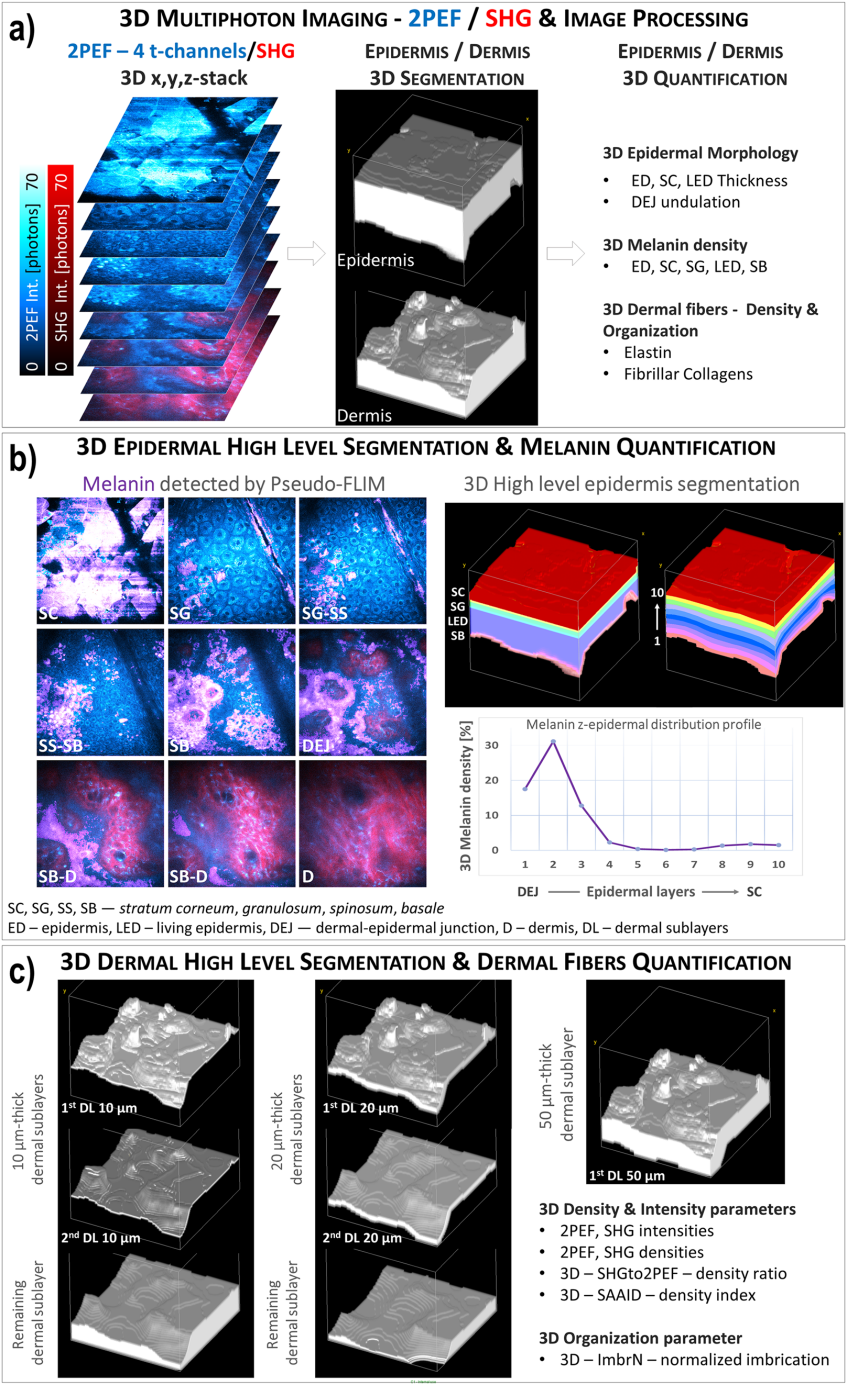
Global analysis process for in vivo 3D multiphoton images allowing 3D skin automatic layers segmentation and constituents quantification. (**a**) Briefly, the first step of global 3D analysis of z-stacks of combined 2PEF-FLIM (4 time channels)/SHG images consists in identifying the epidermal and dermal layers (3D automatic segmentation) and quantifying their morphology (thickness, DEJ 3D-shape), the 3D global melanin density (in epidermis, SC, SG, LED and SB sublayers) and the 3D dermal density and organization of elastin and fibrillar collagens. (**b**) A 3D high level epidermal segmentation is further performed for melanin z-epidermal distribution profile quantification. The 3D z-stacks of melanin masks calculated using the Pseudo-FLIM method (purple color in the 2PEF-cyan hot/SHG-red images), the 3D automatic epidermis segmentation mask and its sublayers (3D high level epidermis segmentation; each color in the 3D reconstruction indicate a different epidermal sublayer) are jointly used in this process. By quantifying the 3D melanin density in 10-thickness normalized epidermal volume sublayers (from 1—DEJ level to 10—SC level), a 3D melanin z-epidermal distribution profile can be obtained. (**c**) A 3D high level dermal segmentation is further performed for elastin and fibrillar collagens quantification. The imaged dermis is divided in 2 thickness normalized (10 or 20 µm thick) dermal volume sublayers that follow the DEJ shape (left and middle 3D reconstructions). A third layer is obtained, the remaining dermal sublayer. The imaged dermis can also be divided into a single 50 µm-thick dermal sublayer that follows the DEJ shape (right 3D reconstruction). Different dermal fibers parameters (intensities, densities, organization) can be calculated in the whole imaged dermal volume or within its sublayers. The data were acquired on the temple face skin area of a young volunteer. The 3D reconstructions were performed with Fiji, 3D Volume viewer.

The global 3D analysis of z-stacks of combined 2PEF-FLIM (4 time channels)/SHG images was performed with MPSTS software^12,13^ to identify skin layers, characterize DEJ 3D-shape and extract quantitative parameters on skin constituents and layers (Fig. 1). Based on mathematical morphology and graph theory, an automatic epidermis/dermis 3D segmentation (Fig. 1a) is computed, followed by a 3D segmentation of SC and living epidermis (LED) sublayers, as previously described^13^.

#### Melanin 3D detection and quantification by Pseudo-FLIM analysis

The z-stack of 2PEF-FLIM (4 time channels) is further processed for melanin detection using Pseudo-FLIM melanin analysis method^32,39,43^ (based on slope analysis of the 2PEF FLIM decay from temporally binned data). The 3D z-stack of melanin masks and automatic epidermis segmentation are jointly used for 3D melanin quantification (Fig. 1b). Global 3D epidermal melanin density is defined as the ratio between the numbers of melanin voxels to epidermal voxels. The 3D melanin density can also be estimated within the epidermal sublayers (e.g. living epidermis LED or a fixed 10 µm layer above the DEJ, mainly corresponding to the basal layer) and within 10 (in this paper) or 12 thickness-normalized epidermal sublayers that follow the 3D shape of the DEJ and SC. Melanin density can be computed in each sublayer and a melanin z-epidermal distribution profile can be extracted (Fig. 1b).

#### Morphological 3D quantification parameters

The 3D morphology of epidermal layers, sublayers and interfaces is characterized by several parameters: mean thickness of SC, LED and epidermis (the thickness is estimated at each pixel location and a mean value is computed for each z-stack) and normalized DEJ area (characterizing DEJ undulation in 3D and defined as the ratio of the DEJ area and its projection on a horizontal plane).

#### Superficial dermis (mainly papillary dermis)—3D quantification of elastin and collagen fibers density

We assess the fibers within the whole imaged dermis (its thickness varies depending on the epidermal thickness) and within different thickness (10, 20 and 50 µm) normalized dermal sublayers (Fig. 1c). The 50 µm normalized thickness value was set to correspond to the thicker dermal sublayer common to all z-stacks in this paper, but this value may vary depending on studies. After applying an intensity threshold to remove the noise and keep the significant 2PEF and SHG pixels, “mainly elastin” and fibrillar collagen masks are obtained. These masks altogether with the dermis segmentation are further processed to estimate the 3D density of elastin and collagen fibers. These parameters are further used to calculate different indexes such as the *3D SHGto2PEF fibrillar collagen to elastin densities ratio* or the *3D SAAID (the SHG-to-AF aging index of dermis)*. The 3D SAAID fibers density index, a straightforward generalization of 2D SAAID, is defined as the ratio between the difference and the sum of fibrillar collagen and elastin fibers densities. A mean value is computed within the superficial dermis layer or normalized dermal sublayers.

#### Superficial dermis (mainly papillary dermis)—3D quantification of elastin and collagen fibers organization

To assess the multimodal 2PEF and SHG signals organization, we measure their overlapping, coincidence at the pixel level by a 3D quantification parameter called normalized imbrication (ImbrN)^13^. This parameter gives an insight into the 3D organization (imbrication, entanglement) of the elastin network compared to the fibrillar collagen network. The 2PEF—elastin and SHG—collagen fibers masks are used to compute this parameter as the ratio between the intersection and union volumes of the 2PEF and SHG masks. A mean value is computed within the superficial dermis layer or normalized dermal sublayers. This parameter can have values between 0 and 1: a large value meaning that the signals are spatially correlated and a low value indicating that they tend to be mutually exclusive.

#### Intensity parameters

The 2PEF and SHG signal intensities can be estimated within the whole z-stack or within the different segmented volumes (layers, normalized sublayers, melanin mask, elastin or fibrillar collagen masks).

For visualization, the combined 2PEF/SHG images in this paper and the 3D epidermal and dermal volume reconstructions were processed with Fiji/ImageJ (W. Rasband, NIH, USA). The 3D elastin fibers networks were reconstructed using Imaris (Bitplane, Zurich, Switzerland).

### Descriptive statistics

The raw data were described using boxplots with the dots and associated density curves displayed on the right side. For the analyses of skin constituent’s variability and robustness of the 3D multiphoton parameters, data from clinical study I with all 4 ROIs were considered. For the analyses of skin changes, based on the results of robustness, data from 2 ROIs per volunteer and per condition were retained for clinical study I. For clinical studies II and III, all available good quality z-stacks were considered (~ 2 ROIs after data quality check). The boxplots were performed with OriginPro (OriginLab, Northampton, Massachusetts, USA).

### Inferential analysis

For the analyses of skin constituent’s variability and robustness of the 3D multiphoton parameters, distributions of the parameters have been compared for 2 vs 3 ROIs and 2 vs 4 ROIs. The homogeneity of variances of the distribution have been compared using the Folded F test (proc TTEST, SAS 9.4) where the F value is calculated as the ratio of the greater of the two variances divided by the lesser of the two variances. Means between ROI’s distributions were compared using a Student t-test.

For the analyses of skin changes, the averaged values based on ~ 2 ROIs per volunteer and per condition have been taken into account for the following analyses.

For clinical study I, comparisons between age and skin color ITA groups have been carried out using an Analysis of Variance (ANOVA) with age, skin color, and their interaction as fixed factors.

For clinical studies II and III, comparisons between age and skin zones have been carried out using a Mixed model of variance analysis with age, skin zone, and their interaction as fixed factors, and subject as a random factor to take into account the within subject correlation between zones.

For the analyses associated to aging difference in the z-dermal distribution, comparisons between age and dermal sub-layers by skin zone have been carried out using a Mixed model of variance analysis with age, dermal sub-layer and their interaction as fixed factors, and subject as a random factor to take into account the within subject correlation between dermal layers.

For all models, comparisons have been performed using contrasts with both t-tests and Cohen-D effect size (ES). Cohen-D is defined as the ratio of within group difference to the squared root of the variance of the sum of random parameters in the model. It gives an idea of the strength of the modifications observed between groups. The effect size depends only on the underlying population parameters, not on the sample size as the *p*-value and allows meaningful comparison between different studies outcomes. The criteria for ES interpretation of multiphoton parameters have been built from contrasts clearly relevant in the study context^41,42^: ES very strong [1.3–Inf], ES strong [0.8–1.3], ES moderate [0.5–0.8], ES weak [0.3–0.5], ES very weak [0–0.3] and no effect if ES = 0.

Inference tests have been carried out using SAS 9.4 (SAS Institute Inc., Cary, NC, USA). The significance threshold for comparisons was set at 5% two-sided. The studies being exploratory, *p*-values have not been adjusted for multiplicity testing.

## Results

To identify human skin changes with aging that could be non-invasively evidenced with multiphoton imaging, we performed three clinical trials (see Materials and methods) on ventral and dorsal forearms and face temple areas of European origin female volunteers. Images were acquired with two different CE-medically approved multiphoton systems (DermaInspect®—study I and II and MPT*flex™*—study III) in equivalent imaging conditions except for slight differences in the field of view (FoV), pixel dwell times and global systems’ sensitivity. In the following, we will firstly address the question of skin constituent’s intrinsic variability and robustness of 3D multiphoton parameters for skin assessment. Secondly, we will present the multiphoton multiparametric 3D quantification results of in vivo human skin changes with aging on forearm and face skin areas.

### Intrinsic skin variability and robustness of 3D multiphoton quantification parameters for skin assessment

Normal human skin has an intrinsic variability in its morphology and in the distribution, density and organization of its native constituents that can be visualized at different scales. Quantifying human skin with multiphoton imaging or in general with other methods implies combining the effects of both skin intrinsic variability and method repeatability (minimal effect).

**Figure 2:**
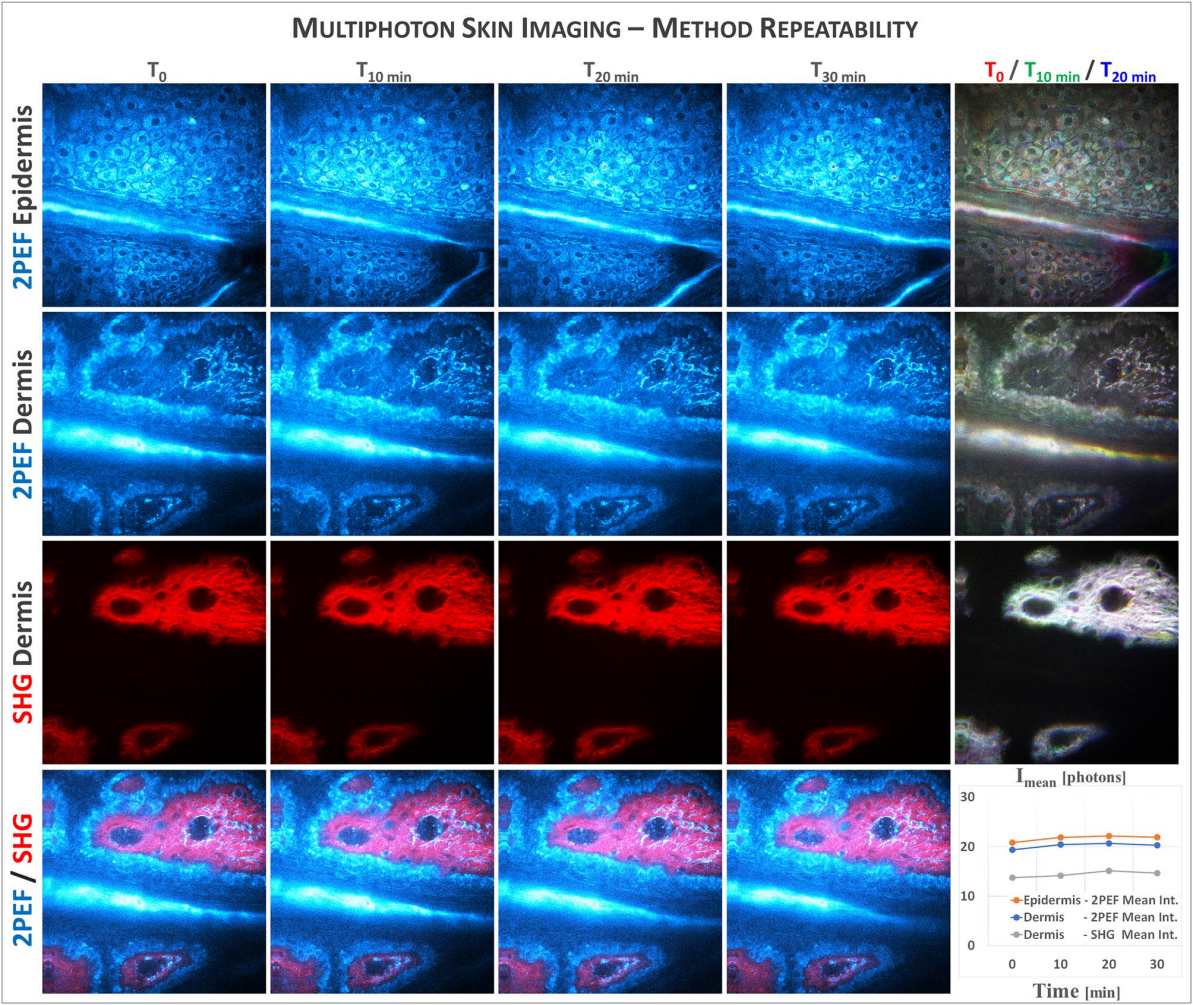
Multiphoton 3D imaging method repeatability—live imaging of human ventral forearm skin recorded every 10 min. Multiphoton 2PEF, SHG and combined 2PEF (cyan hot color)/SHG (red color) images of epidermis and dermis are extracted from a z, t-stack acquired every 2 µm from the skin surface to the dermis and respectively every 10 min within the same skin region and with identical experimental conditions. The right panel images, a combination of images acquired at t = 0 min (red color), t = 10 min (green color) and t = 20 min (blue color) allow observing the time frames colocalization (white when perfect colocalization). The graph shows very slight changes overtime in the mean 2PEF and SHG signals intensity within epidermal and dermal layers, due to slight volunteer movements.

We first verified the multiphoton 3D imaging repeatability and confirmed the minimal influence of the imaging system itself on the skin multiphoton signals, by performing a live imaging experiment where 3D x,y,z-stacks were acquired every 10 min in the same imaging conditions and on the same region of the ventral forearm skin (Fig. 2). One can clearly identify the same epidermal and dermal structures over time, with a good overlap as visualized on the time frames colocalization images and minimal variations in the mean 2PEF and SHG signals intensities (Fig. 2, graph), mainly due to slight volunteer movements. Thus, the variations between the different skin ROIs will essentially depend on the skin intrinsic variability.

In general, in a clinical study different volunteers are included and for each volunteer and experimental condition one can measure one up to several regions of interest. In our studies, given the long image acquisition times (7–10 min/3D z-stack), we often acquire a maximum of 2 or 3 ROIs per volunteer per condition.

To address the question of intrinsic skin variability impact on multiphoton parameters significance and evaluate their robustness for skin assessment, we performed a clinical trial on 20 young (30–40 year-old) and 20 aged (55–65 year-old) European origin volunteers (women and men) with “light” and “tan” skin forearm color (study I). We acquired a maximum of 4 ROIs per volunteer and quantified the 3D mean values of different multiphoton parameters.

**Figure 3:**
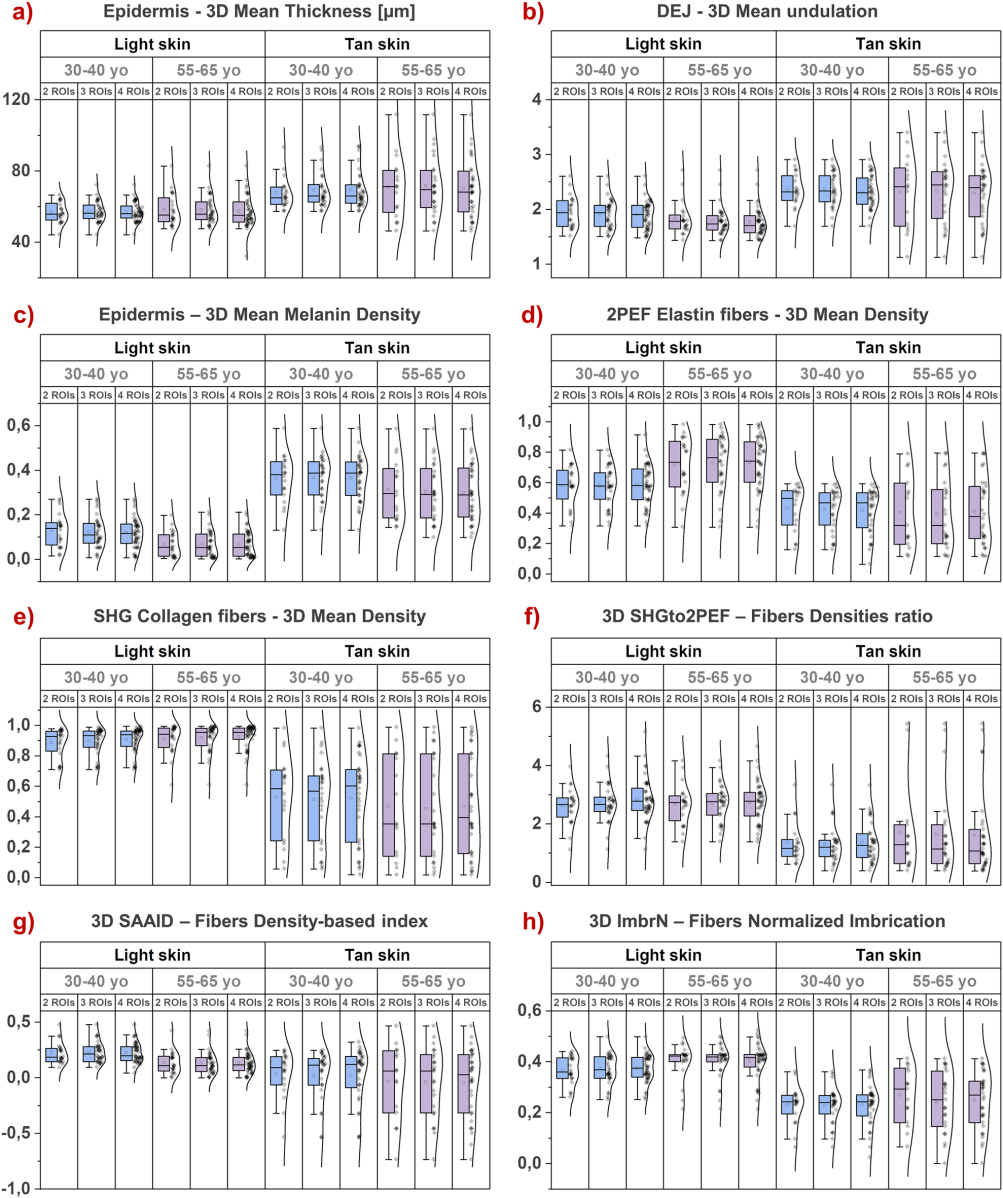
Intrinsic skin variability and robustness of multiphoton 3D quantification parameters for skin assessment. Multiphoton evaluation of ventral forearm skin aging (30–40 vs 55–65 year-old) and color (“light” vs “tan”) differences. (**a**) 3D mean epidermal thickness; (**b**) Mean 3D DEJ undulation; (**c**) 3D Mean melanin density in global epidermis; 3D density of (**d**) 2PEF pixels (corresponding mainly to elastin fibers) and (**e**) SHG pixels (collagen fibers) in a 50-µm thickness normalized dermal sublayer following the DEJ shape. These two parameters are jointly used to calculate (**f**) the 3D SHGto2PEF collagen to elastin fibers densities ratio and (**g**) the 3D SAAID density index (the SHG-to-AF aging index of dermis); (**h**) 3D normalized imbrication (ImbrN) of the elastin and collagen fibers networks within the dermal sublayer. The raw data are expressed as box plots with fences, mean and median (—). All ROIs values and their distribution (dots () and histogram) are shown on the right side of the boxplot. Overlapping values are highlighted in black (♦). Each box plot includes the data from 10 volunteers and 2, 3 or respectively 4 ROIs acquired for each volunteer. The statistical analysis results (*p*-values) of the homogeneity tests for variances and means between the different ROIs groups are given in Table S1.

Figure 3 shows the effects of the number of measurements (ROIs) on several multiphoton skin quantification parameters estimated in 3D: epidermal thickness (Fig. 3a), DEJ undulation (Fig. 3b), melanin density in global epidermis (Fig. 3c) and, within the 50 µm-thick dermal sublayer following the DEJ shape, elastin fibers density (Fig. 3d), collagen fibers density (Fig. 3e) and their combined SHGto2PEF densities ratio (Fig. 3f), SAAID density index (Fig. 3g) and ImbrN normalized imbrication (Fig. 3h). For all multiphoton parameters, we evidenced an overlap of data boxplots and distributions calculated with 2, 3 and respectively 4 ROIs per volunteer. Homogeneity tests for variances and mean comparisons showed no differences between the ROIs’ groups, all *p*-values being very far from the significant threshold (Table S1).

Altogether, these results indicate that in vivo multiphoton normal human skin studies with only 2 ROIs per zone per volunteer are a good compromise in assessing 3D skin constituents compared to acquiring 3 or 4 ROIs per zone per volunteer.

### Aging differences in 3D skin morphology

**Figure 4:**
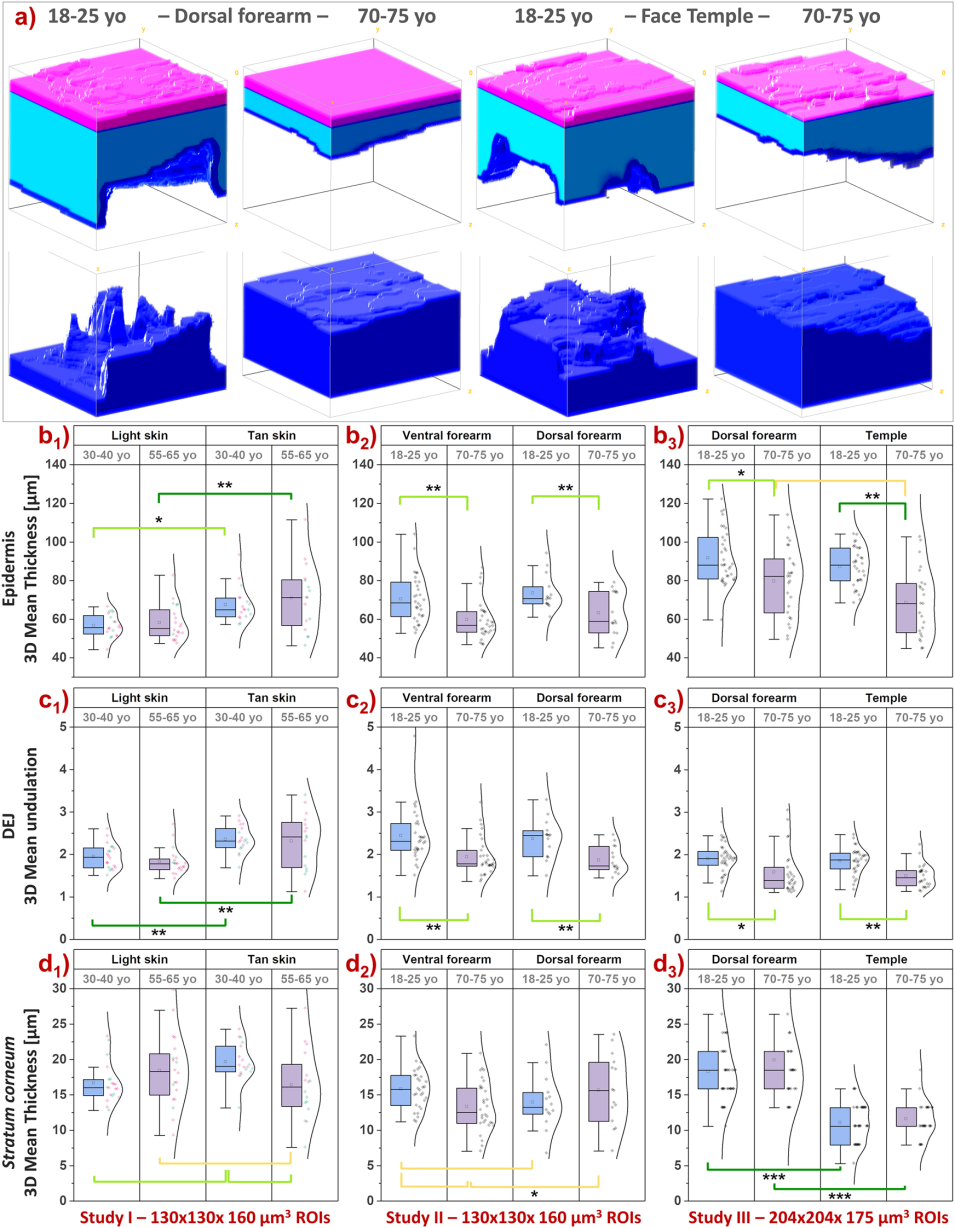
Multiphoton—Aging differences in 3D skin morphology of ventral and dorsal forearms and face temple area. Data from 3 clinical trials: Study I acquired with DermaInspect on ventral forearm (30–40 vs 55–65 year-old; “light” ITA vs “tan” ITA skin color groups); Study II acquired with DermaInspect on ventral and dorsal forearms (18–25 vs 70–75 year-old); Study III acquired with MPT*flex* on dorsal forearm and face temple area (18–25 vs 70–75 year-old). (**a**) 3D reconstructions of epidermal and dermal volumes of dorsal forearm and face temple area of a young and respectively old volunteers with epidermal thickness and DEJ undulation values close to the mean values of the study III groups. (**b1**–**b3**) Mean epidermal thickness; (**c1**–**c3**) Mean 3D DEJ undulation; (**d1**–**d3**) Mean *stratum corneum* thickness. The ventral forearm data in b2 and c2 are adapted from Decencière et al*.* Skin Res Technol 19, 115–124, 2013 (Fig. 6a,b^13^). The raw data are expressed as box plots with fences, mean and median (—). All ROIs values and their distribution (dots () and histogram) are shown on the right side of the boxplot. Overlapping values are highlighted in black (♦). In study I, the pink  and green  data points correspond to respectively women and men volunteers. The mean and median values with their errors are given in Table S2. All the statistical *p*-values and ES parameters are given in Table S3. Statistically significant *p*-values: *≤ 0.05; **≤ 0.01, ***≤ 0.001. The colored brackets indicate the ES—effect size (dark green—very strong [1.3–Inf], light green—strong [0.8–1.3] and yellow—moderate [0.5–0.8]). Only *p*-values associated with moderate to very strong ES are shown.

As illustrated by the 3D reconstructions of epidermal and dermal volumes acquired within dorsal forearm and face temple areas (Fig. 4a), multiphoton microscopy can evidence changes in both epidermal thickness and DEJ undulation (dermal surface) with aging. We quantified three morphological parameters: 3D mean epidermal thickness (Fig. 4b1–b3), 3D mean DEJ undulation (Fig. 4c1–c3) and 3D mean *stratum corneum* thickness (Fig. 4d1–d3). The highest aging modulations are observed between 18 and 25 and 70–75 year-old groups on all areas with an expected thinning of the epidermis and a flattening of the DEJ in older volunteers (studies II and III, Fig. 4b2,b3,c2,c3) making them strong markers of intrinsic aging. Between closer and less extreme age groups (30–40 vs 55–65, study I, Fig. 4b1,c1), we observed no changes in these parameters. However, the higher epidermal thickness and more undulated DEJ in “tan” skin ITA group, better protected against chronic exposure compared to “light” skin ITA group, suggest that these parameters are also, probably to a lesser extent, modulated by photoaging. The higher decrease in epidermal thickness on temple area with aging, more exposed as compared to dorsal forearm, is a second argument in this sense (study III).

Regarding the 3D mean thickness of the SC layer, the main difference is observed between face and forearm, with a clear thinner SC layer on the temple (Fig. 4d3). Total epidermal thickness being equivalent between these two zones, it results in a thicker living epidermis on the face, probably due to an intrinsic structural difference. A thinner SC was also measured in “light” compared to “tan” skin ITA phototypes in the 30-40yo age group (Fig. 4d1). With aging, on ventral forearm, a small decrease in SC layer thickness was observed between the 18–25 and 70–75 year-old groups (study II, Fig. 4d2 left) and between the 30–40 and 55-65yo groups with “tan” skin ITA phototypes (Fig. 4d1 right). Given the minimal (Fig. 4d2 left) or absence (Fig. 4d2 right, d3) of aging changes in SC thickness, aging modulations of epidermal thickness on forearm and face areas are mainly due to that of the living epidermis.

### Skin color and aging differences in melanin’s 3D density and z-epidermal distribution

**Figure 5:**
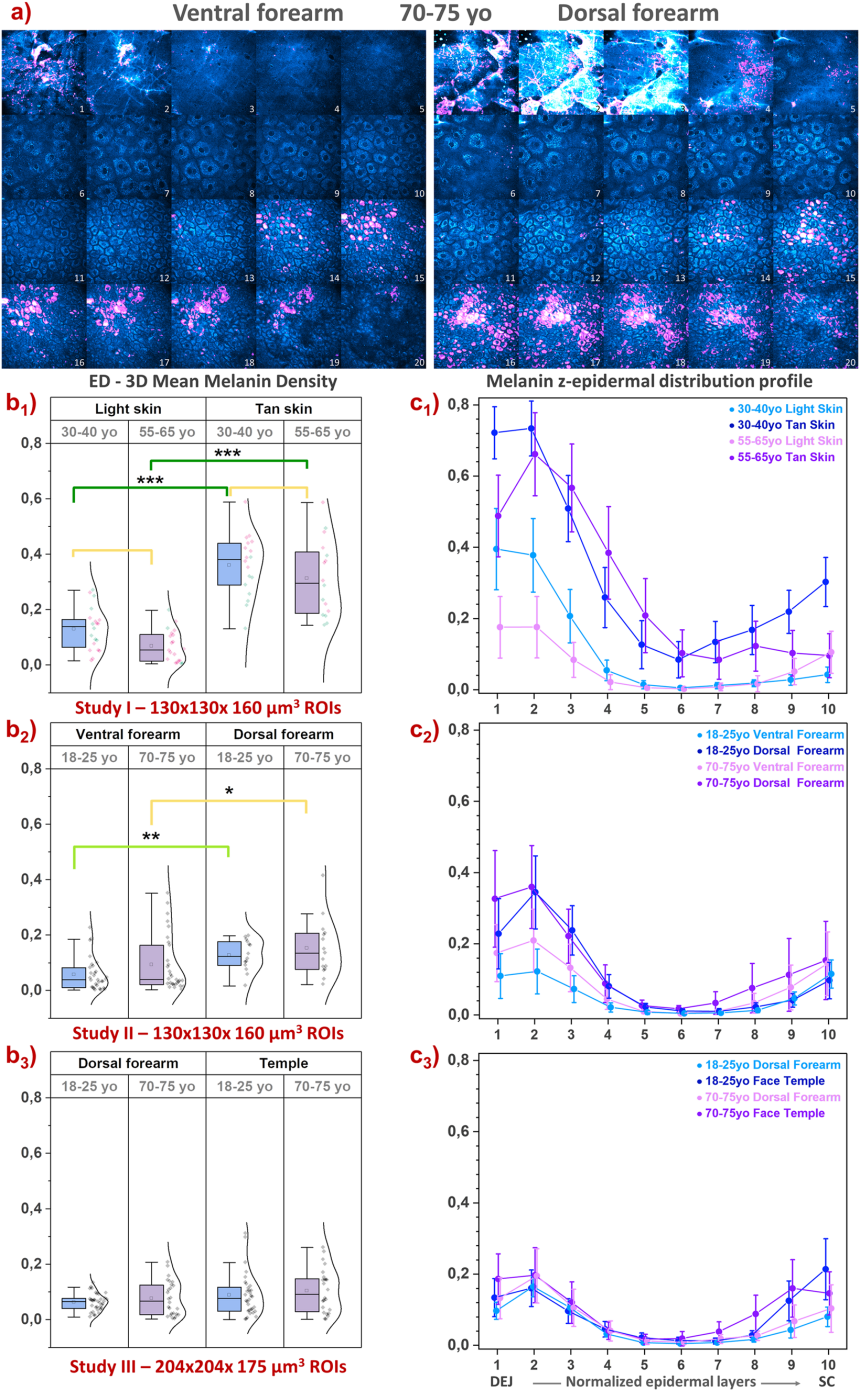
Multiphoton — Skin color and aging differences in melanin 3D density and z-epidermal distribution profile. Data within the global epidermis from 3 clinical trials: study I acquired with DermaInspect on ventral forearm (30–40 vs 55–65 year-old; “light” ITA vs “tan” ITA skin color groups); study II acquired with DermaInspect on ventral and dorsal forearms (18–25 vs 70–75 year-old); study III acquired with MPT*flex* on dorsal forearm and face temple area (18–25 vs 70–75 year-old). (**a**) Mosaic representation of a z-substack of 20 in vivo multiphoton images (labels 1–20) acquired every 2.346 µm from the *stratum corneum* to the basal layer on ventral and respectively dorsal forearm skin of an old volunteer (study II). 2PEF intensity is shown in cyan hot color and Pseudo-FLIM melanin mask pixels in purple. High 2PEF signal intensities appear in white color. (**b1**–**b3**) 3D Mean melanin density in epidermis (ED); (**c1**–**c3**) 3D Melanin density z-epidermal distribution profiles (mean 3D melanin density estimated in 10 thickness-normalized epidermal layers from 1—DEJ dermal–epidermal junction level to 10—SC *stratum corneum* level). The data in b2 and c2 are adapted from Pena et al*.* Sci. Rep. 12, 1642, 2022 (Fig. S7^32^). The b1, b2 and b3 raw data are expressed as box plots with fences, mean and median (—). All ROIs values and their distribution (dots () and histogram) are shown on the right side of the boxplot. Overlapping values are highlighted in black (♦). In study I, the pink  and green  data points correspond to respectively women and men volunteers. The (**c1**–**c3**) data are expressed as mean ± 95% confidence intervals of the mean. The mean and median values with their errors are given in Table S2. All the statistical *p*-values and ES parameters are given in Table S3. Statistically significant *p*-values: *≤ 0.05; **≤ 0.01, ***≤ 0.001. The colored brackets indicate the ES—effect size (dark green—very strong [1.3–Inf], light green—strong [0.8–1.3] and yellow—moderate [0.5–0.8]). Only *p*-values associated with moderate to very strong ES are shown.

Using quantification parameters such as the 3D melanin density in global epidermis and its z-epidermal distribution, we assessed melanin modulations with skin color (“light” vs “tan” ITA skin groups), anatomical skin zone and aging in the three clinical trials (Fig. 5). As expected, differences in melanin density were mainly evidenced between “light” and “tan” ITA groups (study I, Fig. 5b1) and between less exposed and sun exposed skin areas i.e. ventral and dorsal forearm sides (study II, Fig. 5a,b2), with no difference between dorsal forearm and face temple area which have a close chronic exposure (study III, Fig. 5b3).

The melanin density z-distribution profiles allow refining the analysis. It highlights higher differences in the basal and supra-basal layers of “light” skin between the two groups of age in study I (Fig. 5c1) and accumulation of melanin in basal and supra-basal layers with aging mainly on ventral forearm (Fig. 5c2), making this parameter a potential marker of photoaging. That remains to be confirmed since no clear differences of epidermal melanin distribution was evidenced in study III (Fig. 5c3, see “Discussion”).

### Elastin—3D density and z-dermal distribution changes with aging

**Figure 6:**
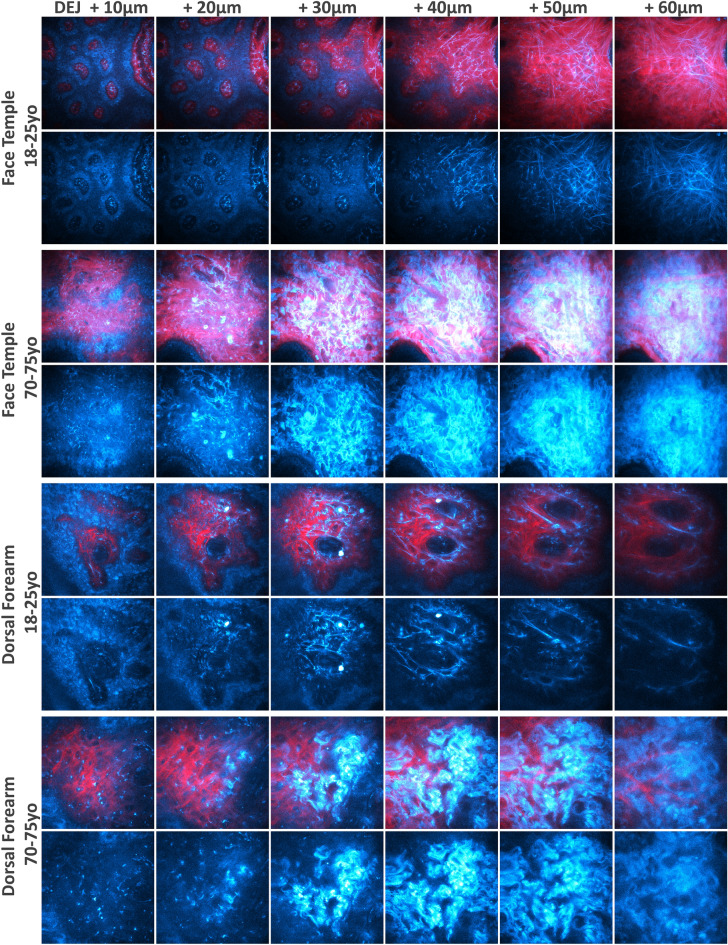
In vivo multiphoton images of dorsal forearm and face temple areas showing elastin aging changes. Representative multiphoton 2PEF (cyan hot—cells and elastin) and SHG (red—fibrillar collagens) images acquired at different depths from the DEJ interface of young (18–25 year-old) and old (70–75 year-old) volunteers. (top) face temple (clinical study III, MPT*flex*, 204 × 204 µm^2^); (bottom) dorsal forearm (clinical study II, DermaInspect, 130 × 130 µm^2^). In young volunteers, elastin 2PEF signal maps the distribution of thin and mostly straight elastic fibers, whereas in old volunteers, thicker and more undulated elastic fibers appear along with abnormal deposits of elastin referred to as solar elastosis.

**Figure 7:**
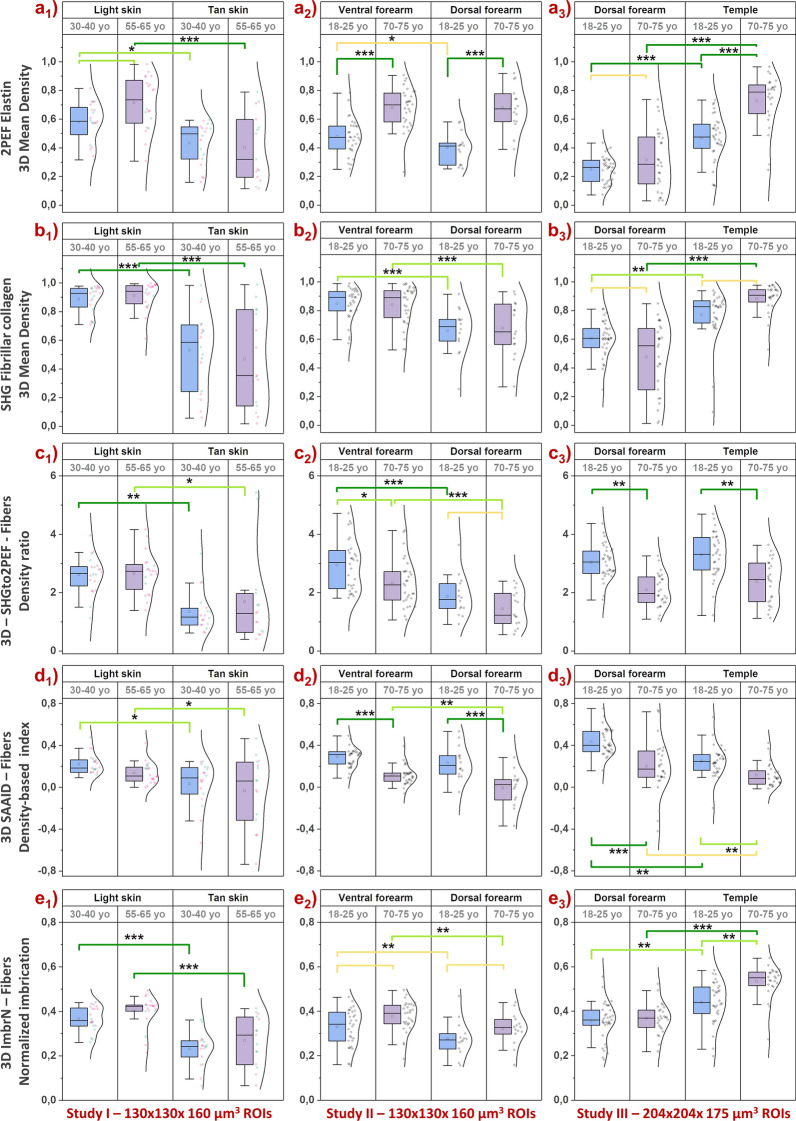
Multiphoton — Aging differences in the 3D density and organization parameters of elastin and collagen fibers networks. Data within the 50-µm thick dermal sublayer from 3 clinical trials: study I acquired with DermaInspect on ventral forearm (30–40 vs 55–65 year-old; “light” ITA vs “tan” ITA skin color groups); study II acquired with DermaInspect on ventral and dorsal forearms (18–25 vs 70–75 year-old); study III acquired with MPT*flex* on dorsal forearm and face temple area (18–25 vs 70–75 year-old). (**a1**–**a3**) 3D Mean 2PEF elastin fibers density; (**b1**–**b3**) 3D Mean SHG collagen fibers density; (**c1**–**c3**) 3D Mean SHGto2PEF fibrillar collagen to elastin densities ratio; (**d1**–**d3**) 3D Mean SAAID fibrillar collagen and elastin density-based index; (**e1**–**e3**) 3D Mean ImbrN fibrillar collagen and elastin normalized imbrication index. The raw data are expressed as box plots with fences, mean and median (—). All ROIs values and their distribution (dots () and histogram) are shown on the right side of the boxplot. Overlapping values are highlighted in black (♦). In study I, the pink  and green  data points correspond to respectively women and men volunteers. The mean and median values with their errors are given in Table S2. All the statistical *p*-values and ES—effect sizes parameters are given in Table S3. Statistically significant *p*-values: *≤ 0.05; **≤ 0.01, ***≤ 0.001. The colored brackets indicate the ES—effect size (dark green—very strong [1.3–Inf], light green—strong [0.8–1.3] and yellow—moderate [0.5–0.8]). Only *p*-values associated with moderate to very strong ES are shown.

As shown in the multiphoton images in Fig. 6, the greatest changes appearing with aging in the superficial dermis are in the elastin network. Except from a few cells (e.g. fibroblasts, blood cells, etc.), the dermal 2PEF signal mainly arises from elastin which can be found within both the elastin fibers and the abnormal elastin deposits (solar elastosis). We quantified the 3D mean density of elastin within a 50-µm thick dermal sublayer following the shape of the DEJ (Fig. 7a1–a3).

Anatomical site differences in the 3D elastin density were evidenced in young (18–25 year-old) volunteers between the dorsal forearm (lowest values) and ventral forearm and face temple areas (highest values) and in middle age (30–40 and 55–65 year-old) volunteers between “light” and “tan” ITA groups. With aging, the most obvious changes were detected between the extreme age groups (18–25 vs 70–75 year-old, studies II and III, Fig. 7a2,a3) with a clear increase in elastin density in older subjects in all areas: face, ventral, and dorsal forearms, although less obvious in study III on this last area. In study III, the difference was greater in the most exposed area (face vs forearm, Fig. 7a3), making this parameter a marker of both chrono- and photo-aging. Moreover, between age groups closer to each other (30–40 vs 55–65 year-old, study I, Fig. 7a1) this difference was only found in the lightest skin subgroup, which is consistent with a better dermal protection afforded by higher epidermal melanin density. The measurement of the real contribution of photoaging in the variations of this parameter during global aging would require a comparison with a totally photoprotected area, which was not planned in these studies.

**Figure 8:**
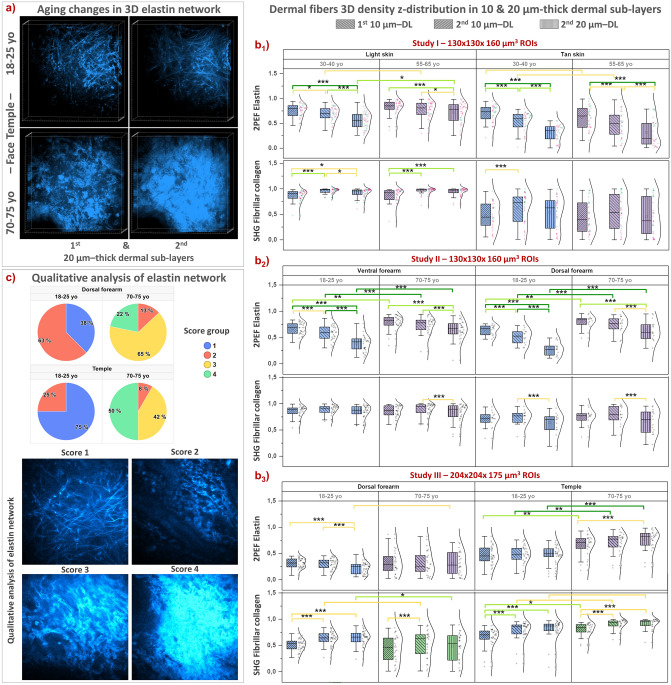
Multiphoton — Aging differences in elastin network organization and in the z-dermal distribution of elastin and collagen fibers 3D density. Data from 3 clinical trials and within the 1st 10 µm, 2nd 10 µm and 2nd 20 µm thickness normalized dermal sublayers (DL) that follow the DEJ shape: study I acquired with DermaInspect on ventral forearm (30–40 vs 55–65 year-old; “light” ITA II vs “tan” ITA IV skin color groups); study II acquired with DermaInspect on ventral and dorsal forearms (18–25 vs 70–75 year-old); study III acquired with MPT*flex* on dorsal forearm and face temple area (18–25 vs 70-75 year-old). (**a**) 3D reconstructions of elastin network within the 1st and 2nd 20 µm thick DL of young and old face temple areas. (**b1**–**b3**) z-dermal distribution profiles of 3D mean 2PEF elastin density and 3D mean SHG collagen fibers density; (**c**) Qualitative analysis of elastin network organization on dorsal and face temple areas (study III) with (top) elastin scores frequency and (bottom) representative 2PEF elastin intensity images for each score. The data are expressed as box plots with fences, mean and median (—). All ROIs values and their distribution (dots () and histogram) are shown on the right side of the boxplot. Overlapping values are highlighted in black (♦). In study I, the pink  and green  data points correspond to respectively women and men volunteers. All the statistical *p*-values and ES—effect sizes parameters are given in Table S4. Statistically significant *p*-values: *≤ 0.05; **≤ 0.01, ***≤ 0.001. The colored brackets indicate the ES—effect size (dark green—very strong [1.3–Inf], light green—strong [0.8–1.3] and yellow—moderate [0.5–0.8]). Only *p*-values associated with moderate to very strong ES are shown.

As illustrated in Fig. 6 and in the 3D reconstructions of elastin network of face temple superficial dermis (Fig. 8a), the elastin density seems to vary with z-dermal depth. Quantification results of its z-dermal distribution within 10 and 20 µm thickness normalized dermal sublayers is shown in Fig. 8b1–b3 (the data in the remaining profound dermal sublayer were not considered given its variable thickness and sometimes lower signal intensity mainly when imaging thick epidermal areas). In younger (18–25 and 30–40 year-old) volunteers, we found no changes in elastin density with z-depth on the temple area, but a progressive decrease on both forearm sides. Elastin’s accumulation with aging modifies these z-distributions towards a less pronounced decrease on forearm and a very slight increase on face temple area of older subjects. The highest aging differences in elastin density were measured in the 2nd 10 µm and 2nd 20 µm thick dermal sub-layers.

Aging changes in elastin network are also structural. To assess elastin network’s organization, we performed a qualitative analysis on face temple and dorsal forearm areas, using 4 scores: *score 1*—thin and straight elastic fibers, *score 2*—thin and wavy fibers, *score 3*—thicker, wavy and fragmented fiber bundles and *score 4*—pattern of very dense, compact elastin network (Fig. 8c bottom). For each z-stack, a global representative score of the elastin network was chosen.

This qualitative analysis (Fig. 8c top) allowed evidencing that in young volunteers (18–25 year-old), the elastin network is mostly organized into *score 1* (63% dorsal forearm; 75% face temple) and to a lesser extent into *score 2* (38% dorsal forearm; 25% temple). In older volunteers, the elastin network can be qualified as a mixture of *score 2* (13% dorsal forearm; 8% temple), *score 3* (65% dorsal forearm; 42% temple) and *score 4* (22% dorsal forearm; 50% temple). To be noted, in young volunteers, score 1 is more frequent on face temple area compared to dorsal forearm, whereas in older volunteers, score 3 is more frequent on dorsal forearm and score 4 on face temple area, suggesting an initial difference in the elastin network, more stretched on the temple area than on the dorsal forearm, and a more severe elastosis on the more chronically exposed skin site with aging.

### Collagen fibers—3D density and z-dermal distribution changes with aging

Using the SHG signal of fibrillar collagens, we quantified the 3D mean density of collagen fibers within a 50-µm thick dermal sublayer that follows the shape of the DEJ (Fig. 7b1–b3).

As for elastin density, anatomical site differences in the 3D fibrillar collagen density were evidenced in young (18–25 year-old) volunteers between the dorsal forearm (lowest values) and ventral forearm and temple areas (highest values) (studies II and III, Fig. 7b2,b3). With aging, superficial dermis showed no significant changes in fibrillar collagens density on ventral and dorsal forearms, but a slight increase was highlighted on face temple area (Fig. 7b3).

On ventral forearm, we measured differences between “light” and “tan” skin ITA groups (study I, Fig. 7b1), that could be explained either by an intrinsic difference between the two volunteers groups or by the attenuation of the SHG signal by the higher epidermal melanin density and thicker SC and epidermis in the more pigmented group (see “Discussion”). The SHG mean intensity is indeed smaller in “tan” versus “light” skin ITA groups (Fig. S1b1) and shows a smaller data variability than the SHG density parameter (Fig. 7b1). We calculated the correlations between the dermis SHG mean signal intensity and different parameters such as the epidermal thickness and the melanin density (Fig. S2, ggPairs plots with Pearson correlation coefficients). We found a moderate negative correlation of the SHG signal intensity with the melanin density at the global level (− 0.693) and within the 55–65 year-old “tan” skin color group (− 0.661). The same level of negative correlation was also found with epidermal thickness parameter at the global level (− 0.617), but not within the individual age and skin color groups.

We also characterized the z-dermal distribution of fibrillar collagen density, in the same thickness normalized dermal sublayers as for elastin density (Fig. 8). Globally, for all age groups, we observed no change in collagen fibers density with z-dermal depth on ventral forearm (study I, Fig. 8b1 and study II, Fig. 8b2), nor on dorsal forearm (study II, Fig. 8b2 and study III, Fig. 8b3). On the contrary, on the face temple, the fibrillar collagen density seems to slightly increase with z-depth for both age groups (study III, Fig. 8b3) and to be higher in the older group for all sublayers.

### Aging differences in 3D collagen/elastin density based SHGto2PEF ratio and SAAID index

Two indexes are calculated by combining the parameters of elastin and collagen fibers 3D densities: the 3D SHGto2PEF fibers densities ratio (Fig. 7c1–c3) and the 3D SAAID fibers density-based index (Fig. 7d1–d3), also called the aging index of dermis. These parameters allow summarizing the changes in both elastin and fibrillar collagen densities, but their interpretation requires the knowledge of the variations in the original parameters.

For both parameters, main changes are observed between extreme age groups on every area (study II, Fig. 7c2,d2 and study III, Fig. 7c3,d3) making their decrease a good marker of chrono-aging. The decrease observed between ventral and dorsal forearms both in young and old groups shows that they are also influenced by photo-aging (study II, Fig. 7c2–d2). Since these parameters are ratios, they globally translate a higher elastin density in older (Fig. 7a), but they can also reflect smaller, positive/negative modulations in both elastin and collagen (Fig. 7b3). Indeed, despite a greater photo-exposure, these parameters are comparable on the temple and the dorsal forearm, due to the structurally greater density of collagen on the face which compensates for the higher elastosis. In the same way, on dorsal forearm (study III, Fig. 7c3), these indexes reflect an aging decrease while elastin and fibrillar collagen densities show only small changes, essentially broader data distributions with aging. This proves the importance of assessing separately the elastin and fibrillar collagens networks and their aging modulations.

Analysis of the z-dermal distributions of the 3D SHGto2PEF fibers densities ratio (Fig. S3a–c) and the 3D SAAID fibers density-based index (Fig. S4a–c) globally shows z-layers differences (progressive increase with z-depth) in young volunteers, more attenuated with aging. For both indexes, the highest aging difference was measured in the 2nd 20 µm thick dermal sub-layer.

### Aging differences in 3D elastin/fibrillar collagen normalized imbrication index ImbrN

We characterized the proximity or the imbrication of the elastin and collagen fibers networks at the pixel level using the normalized imbrication index.

Anatomical site differences in the 3D mean ImbrN index were evidenced in middle age (30–40 and 55–65 year-old) volunteers between “light” and “tan” ITA groups (lowest values) (study I, Fig. 7e1), in young (18–25 year-old) and old (70–75 year-old) volunteers between the ventral forearm and dorsal forearm (lowest values) (study II, Fig. 7e2) and between dorsal forearm and face temple areas (highest values) (study III, (Fig. 7e3).

An aging increase in the 3D mean ImbrN index was only observed between the extreme age groups (18–25 vs 70–75 year-old) on both forearm sides (study II, Fig. 7e2, moderate ES) and on the face temple area (study III, Fig. 7e3, strong ES), making it a marker of chrono-aging.

It also increases between the less exposed area (forearm) and the more exposed (face) skin areas in study III, due to the high intrinsic fibrillar collagens densities on the face compared to dorsal forearm. Its aging increase is greater on the most exposed area (face vs forearm, Fig. 7a3), due to the higher increase in both elastin and fibrillar collagens, making this parameter a marker of both chrono- and photo-aging on face, since such a photoaging difference was not observed in study II between the 2 forearm sides. The decrease of normalized imbrication between “light” and “tan” skin ITA groups is likely driven by the difference of fibrillar collagens density between them (study I, Fig. 7e1).

Globally, given the 3D fibers densities results (Fig. 7a,b), the changes in this parameter are mainly driven by the 3D elastin density increase which favors a higher proximity of the two networks at the pixel level with aging, and to a lesser extent by the increase in the 3D fibrillar collagen density for the face temple area.

For all skin areas, the z-dermal distribution analysis of the 3D mean ImbrN index (Fig. S5a–c) shows a decrease of this index with z-depth in young volunteers, more attenuated with aging, and highlights the 2nd 20 µm thick dermal sub-layer as being the sublayer characterized by the highest aging difference.

## Discussion and conclusions

After checking the strong repeatability of the technique, we first assessed the robustness of multiphoton quantification parameters that was lacking in the literature. Indeed, knowing the intrinsic variability of human skin and the quite limited field of view of the technique, it was legitimate to ask the question of the number of measurements to be made to correctly estimate skin quantitative parameters.

Normal human skin has effectively a variability in its native constituents that can be evidenced at different scales from dermoscopy, histology to multiphoton and other higher resolution imaging techniques such as transmission electron microscopy. For example, on histological sections, one can clearly see the variations in epidermal thickness, simply because of the DEJ undulation, or the non-regular distribution of melanin in the basal layer. In dermis, variations between papillary and reticular dermis in terms of density of elastin (elastin fibers and elastosis) and collagen fibers, or their thickness and organization are another example. For the epidermis, compared to histology analysis, in which quantification is generally performed on a few 2D ROIs of ~ 200–250 µm epidermal length, in multiphoton 3D imaging the investigated skin volume is higher (e.g., ~ 200 × 200 µm^2^ × epidermal thickness). Despite this still relatively small investigated epidermal volume, we have previously shown that 3D morphological epidermal parameters (thickness and DEJ shape) as well as global 3D epidermal melanin density and its z-epidermal distribution^32^, enable studying various physiological conditions (constitutive and acquired pigmentation^32^, aging^32^, natural UV exposure^42^) or treatment effect (topical retinoic acid and retinol^41,42^ or corticosteroids^24^). Here, we further demonstrated that for in vivo multiphoton human skin studies, our experimental protocol (2 ROIs per zone per volunteer) is robust compared to protocols involving 3 or 4 ROIs per volunteer and relevant in assessing in 3D normal human skin and its aging changes in terms of epidermal thickness, DEJ undulation, melanin density, elastin density, collagen density, SHGto2PEF densities ratio, SAAID density index and ImbrN normalized imbrication.

In this work, the same age groups and, for part, the same skin area were investigated with two different CE-medically approved multiphoton systems (DermaInspect®—study II and MPT*flex™*—study III) and results were very similar. The modulation amplitude of some parameters slightly differs, first as they were not collected at the same time nor on the same volunteers, but also due to differences in the field of view, pixel dwell times and global systems’ sensitivity of the two microscopes. It is logical to consider that the larger FoV (MPT*flex™*) affords a more precise estimation of mainly the mean epidermal thickness, in cases where the DEJ is very undulated, as several dermal papillae can be imaged, depending on their size and shape, and the rete ridges detected. A larger (~ 1.6 × 1.6 mm^2^) skin area sampling was shown to improve the precision of melanin density estimation compared to smaller skin areas, without influencing its average value’ estimation^44^. Large-area based melanin quantification of different skin Fitzpatrick phototypes^44^ shown comparable results to our previous small-area constitutive pigmentation data^32^, albeit differences in melanin detection and experimental protocols. The small-area based 3D multiphoton quantification parameters (epidermal morphology, melanin density and its z-distribution), estimated within the plateau regions of the skin, may not afford absolute measurements but they have proven to be relevant in detecting normal human skin changes occurring physiologically^32,42^ or upon treatment application^24,41,42^. Considering the results in the current work, this statement can be extended to dermal 3D parameters and photo-aging characterization. For melanin assessment, a larger skin area would afford a more precise measurement, but in our opinion would mainly be of interest in assessing melanin heterogeneity in, for example, skin disorders and skin cancers. The influence of large-area sampling on epidermal morphology, dermal elastin and collagen fibers 3D parameters remains to be evaluated.

Regarding the modulation of the studied 3D parameters during aging, the three major facts are the epidermal atrophy, the DEJ flattening and the elastosis in the dermis in agreement with histology data^2^. Moreover, in accordance with the literature^34^, epidermal thinning and the consubstantial DEJ flattening occur late and is only objectified between extreme age ranges. In the dermis, among the combined 3D collagen/elastin density-based parameters of SHGto2PEF ratio, SAAID index and normalized imbrication ImbrN, the last two ones provide the most detailed complementary information, refining the share of photo-aging in the quantification of global aging, which elastin or fibrillar collagens densities alone do not allow. However, the separate assessment of elastin and fibrillar collagens networks is critical for the interpretation of the combined parameters. Moreover, analysis of the z-dermal distribution of these parameters highlighted the 2nd 20 µm thickness normalized dermal sub-layer, that follows the DEJ shape, as exhibiting the highest aging differences. In addition, the qualitative analysis on the high-resolution multiphoton 3D images allows to grade the changes in the conformation of the elastic network with aging and exposure, providing an additional information to its quantification.

Concerning pigmentation characterization, using the 3D melanin global density, we could non-invasively quantify the expected increase with skin color (“tan” > “light” ITA groups), in agreement with previous data on constitutive and acquired pigmentation^32^ and chronic exposure (dorsal > ventral forearms)^32^. In addition to melanin amount quantification, its 3D epidermal distribution allows to objectify differences even when the global density does not vary. In study II, comparing aging (18–25 vs 70–75 year-old) differences between dorsal and ventral forearms, it evidenced a suprabasal accumulation both with age and with chronic exposure. Such modifications in melanin distribution were not evidenced in study III comparing same age groups and areas with different degree of exposure. Thus, this is to be explored in a future study with volunteers with homogeneous constitutive pigmentation and a quantified and comparable degree of sun exposure at inclusion, which was not the case in these two studies.

Regarding studied areas, the major differences evidenced are a thinner *stratum corneum*, a thicker living epidermis and a higher elastin and fibrillar collagens densities on the face temple area compared to the dorsal forearm, both in young and older volunteers, corresponding to intrinsic anatomical differences already reported in histological studies^45,46^. A thinner SC was also reported with confocal reflectance and Raman measurements on cheek (28–54 year-old)^47^. The great interest of the non-invasiveness of in vivo multiphoton imaging is really to be emphasized particularly when concerning the face. Photoaging of the temple area evolves as a function of chronic exposure as measured by the more pronounced increase in elastin density and the slight increase in fibrillar collagens density, the decrease in the 3D SHGto2PEF ratio and 3D SAAID index and the increase in the normalized imbrication 3D ImbrN index. For the area comparisons, due to the intrinsic higher elastin and fibrillar collagens amount on the face and to their aging increase, it is the normalized imbrication 3D ImbrN parameter that allows to refine the share of photoaging in global aging, whereas 3D SAAID do it the best on the forearm. The normalized imbrication parameter 3D ImbrN, measuring the degree of spatial correlation between these two fiber networks, gives an insight into their mutual organization and translates their entanglement. In the context of aging, the modulation of these three dermal parameters is mainly driven by the important modification of elastin density and their interest in other clinical contexts (diseases of the elastic tissue, collagenosis…) remains to be determined.

The study of two groups with different skin color (ventral forearm, study I) draws attention to the necessary precautions when interpreting the quantification of dermal fibrillar networks in this context. Indeed, we clearly observe a lower fibrillar collagens density in the more pigmented group in both age groups. We cannot totally exclude a difference linked to different phototypes, as elastin density was also smaller in the “tan” ITA group, but the subjects are all of European origin and such a difference is surprising and is not in accordance with histological knowledge. Moreover, although to a lesser degree, the same observation can be made between the more pigmented dorsal side and the ventral side of the forearm within the same group (study II). In our opinion, the most likely explanation is an attenuation of the dermis SHG signal by both absorption and scattering phenomena in thicker SC and epidermal areas with increased melanin density, as suggested by the negative correlations. Therefore, studies comparing different groups of skin color should be limited to epidermis, melanin and elastin characterization and comparison of SHG dermal signals restricted within the same phototype or carefully interpreted between anatomical areas with too strong pigmentation differences.

In conclusion, in this work (1) we introduced a multiphoton multiparametric quantification toolbox for 3D skin assessment, (2) studied their robustness in characterizing normal human skin pigmentation and aging changes with respect to skin intrinsic variability and (3) applied these tools to skin aging characterization on different UV exposed areas—ventral and dorsal forearms, face. The three major facts of aging that are epidermal atrophy, the dermal–epidermal junction flattening and dermal elastosis can be non-invasively quantified and compared. Epidermal morphological changes occur late and were only objectified between extreme age groups. Melanin accumulation in suprabasal layers with age and chronic exposure on ventral and dorsal forearms is less known and appears earlier. Superficial dermal aging changes are mainly elastin density increase, with no obvious change in collagen density, reflected by SHGto2PEF ratio and SAAID index decrease and ImbrN index increase on all skin areas. Analysis of the z-dermal distribution of these parameters highlighted the 2nd 20 µm thickness normalized dermal sub-layer, that follows the DEJ shape, as exhibiting the highest aging differences. Moreover, the 3D ImbrN index allows refining the share of photoaging in global aging on face and the 3D SAAID index on forearm, which elastin or fibrillar collagens densities alone do not allow. Photoaging of the temple area evolves as a function of chronic exposure with a more pronounced increase in elastin density, also structurally modified from thin and straight elastic fibers in young volunteers to dense and compact pattern in older ones.

This work also highlights the importance of using different 3D morphology, density and organization parameters to create a global picture of not only the dermal fibers but also of the epidermis morphology and its melanin pigment. This analysis could be completed by other measurements such as the size and number of cells within living epidermis^24,48,49^ or the fibers orientation^17^ estimated up to now only in specific 2D planes. Other organization^12^ parameters could also be implemented to address the fibers networks aging changes in 3D and will be the subject of another paper. Besides skin aging applications, we have previously shown the potential of 3D quantification tools to non-invasively characterize skin constitutive pigmentation^32^ and assess the effects of anti-aging compounds such as retinoic acid and retinol^41,42^. More generally, multiphoton multiparametric 3D skin quantification offers rich spatial information of interest in assessing normal human skin condition and its pathological, external environment or product induced changes.

## Supplementary Information

Supplementary Information.

## Data Availability

The datasets generated during the current work are available from the corresponding author on reasonable request.
