# Supplementary material for: In vivo multiphoton multiparametric 3D quantification of human skin aging on forearm and face
Source: Sci Rep. 2022 Sep 1;12:14863. doi: 10.1038/s41598-022-18657-z (PMC9437074; doi:10.1038/s41598-022-18657-z)
Supplement: Supplementary file 1 — Supplementary Information. [file 41598_2022_18657_MOESM1_ESM.docx]

***In vivo* multiphoton multiparametric 3D quantification
 of human skin aging on forearm and face**

Ana-Maria Pena^1*^, Thérèse Baldeweck^1^, Etienne Decencière^2^, Serge Koudoro^2^, Steeve Victorin^1^, Edouard Raynaud^1^, Blandine Ngo^1^, Philippe Bastien^1^, Sébastien Brizion^1^, Emmanuelle Tancrède-Bohin^3,4*^

^1^L’Oréal Research and Innovation, 1 avenue Eugène Schueller, BP22, 93601, Aulnay-sous-Bois, France.

^2^MINES ParisTech – PSL Research University, Fontainebleau, France.

^3^L’Oréal Research and Innovation, Campus Charles Zviak RIO, 9 rue Pierre Dreyfus, Clichy, France.

^4^Service de Dermatologie, Hôpital Saint-Louis, Paris, France.

*email: [ana-maria.pena@rd.loreal.com](mailto:ana-maria.pena@rd.loreal.com); [emmanuelle.tancrede-bohin@rd.loreal.com](mailto:emmanuelle.tancrede-bohin@rd.loreal.com)

# Supplementary Figures


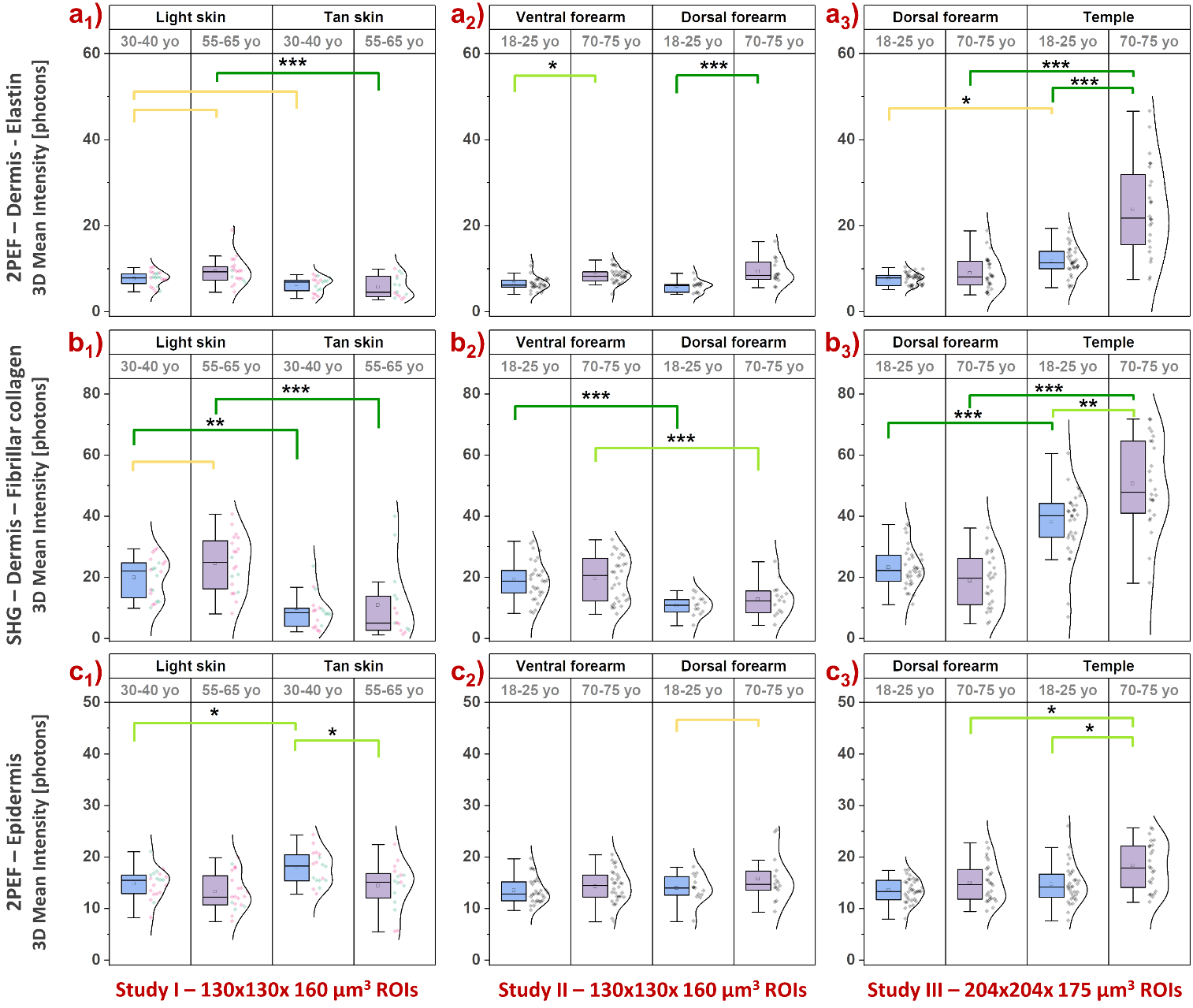


**Figure S 1: Multiphoton – Aging differences in the 3D mean 2PEF and SHG signal intensities of epidermal and dermal layers within ventral and dorsal forearms and face temple areas.** Data from 3 clinical trials: study I acquired with DermaInspect on ventral forearm (30-40 vs 55-65yo; “light” ITA vs “tan” ITA skin color groups); study II acquired with DermaInspect on ventral and dorsal forearms (18-25 vs 70-75yo); study III acquired with MPT*flex* on dorsal forearm and face temple area (18-25 vs 70-75yo). **a1, a2, a3)** 3D Mean 2PEF signal intensity (mainly elastin) and **b1, b2, b3)** 3D Mean SHG signal intensity (fibrillar collagens) within the 50-µm thick dermal sublayer; **c1, c2, c3)** 3D Mean 2PEF signal intensity in global epidermis. The data are expressed as box plots with fences, mean and median (—). All ROIs values and their distribution (dots (♦) and histogram) are shown on the right side of the boxplot. Overlapping values are highlighted in black (♦). The mean and median values with their errors are given in Table S2. All the statistical *p*-values and ES – effect sizes parameters are given in Table S3. Statistically significant *p*-values: *≤0.05; **≤0.01, *** ≤0.001. The colored brackets indicate the ES – effect size (dark green - very strong ]1.3 - Inf], light green - strong ]0.8 - 1.3] and yellow - moderate ]0.5 - 0.8]). Only *p*-values associated with moderate to very strong ES are shown.


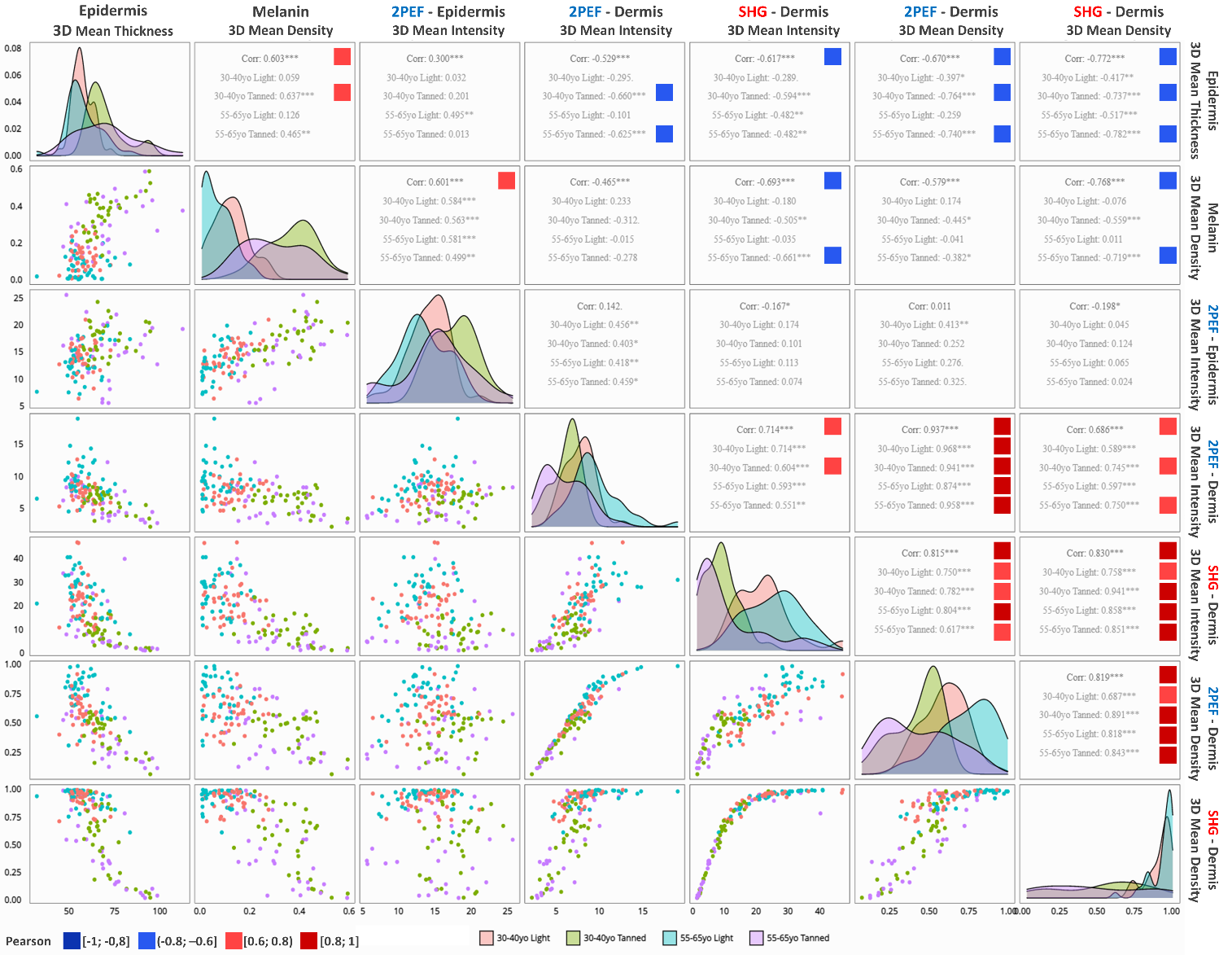


**Figure S 2: Correlation analysis– ggpairs plots allowing to investigate the eventual relationship between the SHG signal intensity and other multiphoton parameters.** Scatter plots of each pair of parameters (epidermal 3D mean thickness, 3D mean melanin density, epidermal 2PEF 3D mean intensity, dermal 2PEF 3D mean intensity and density, dermal SHG 3D mean intensity and density) are shown on the left part of the figure. Their respective global and at the group level Pearson correlation coefficients are shown on the right along with the *p*-values (*≤0.05; **≤0.01, *** ≤0.001) indicating the significance correlation level. An absolute value of the Pearson correlation coefficients between 0.6 and 0.8 indicates a strong and between 0.8 and 1 a very strong correlation. The positive (red) and negative (blue) Pearson correlation coefficients with strong and very strong correlations are highlighted by the blue and red squares. (The parameters’ distributions by groups are shown on the diagonal. Data from clinical study I acquired with DermaInspect on ventral forearm (30-40 vs 55-65yo; “light” ITA vs “tan” ITA skin color groups); All 4 ROIs values per volunteer are shown.


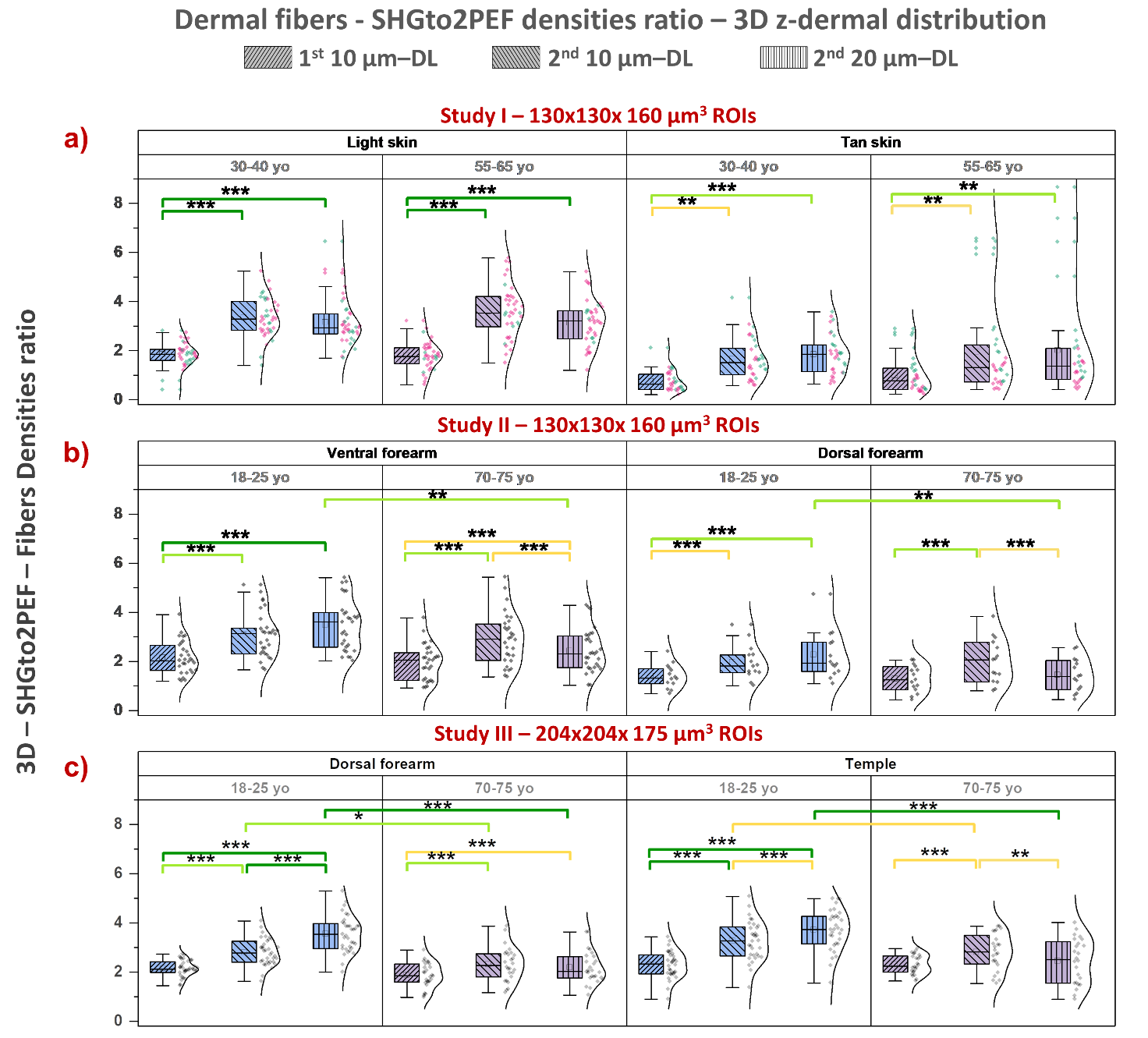


**Figure S 3: Multiphoton – Aging differences in the z-dermal distribution of the 3D – SHGto2PEF fibers densities ratio elastin and collagen fibers 3D density.** Data from 3 clinical trials and within the 1^st^ 10 µm, 2^nd^ 10 µm and 2^nd^ 20 µm thickness normalized dermal sublayers (DL) that follow the DEJ shape: **a)** study I acquired with DermaInspect on ventral forearm (30-40 vs 55-65yo; “light” ITA vs “tan” ITA skin color groups); **b)** study II acquired with DermaInspect on ventral and dorsal forearms (18-25 vs 70-75yo); **c)** study III acquired with MPT*flex* on dorsal forearm and face temple area (18-25 vs 70-75yo). The data are expressed as box plots with fences, mean and median (—). All ROIs values and their distribution (dots (♦) and histogram) are shown on the right side of the boxplot. Overlapping values are highlighted in black (♦). In study I, the pink ♦ and green ♦ data points correspond to respectively women and men volunteers. All the statistical *p*-values and ES – effect sizes parameters are given in Table S4. Statistically significant *p*-values: *≤0.05; **≤0.01, *** ≤0.001. The colored brackets indicate the ES – effect size (dark green - very strong ]1.3 - Inf], light green - strong ]0.8 - 1.3] and yellow - moderate ]0.5 - 0.8]). Only *p*-values associated with moderate to very strong ES are shown.


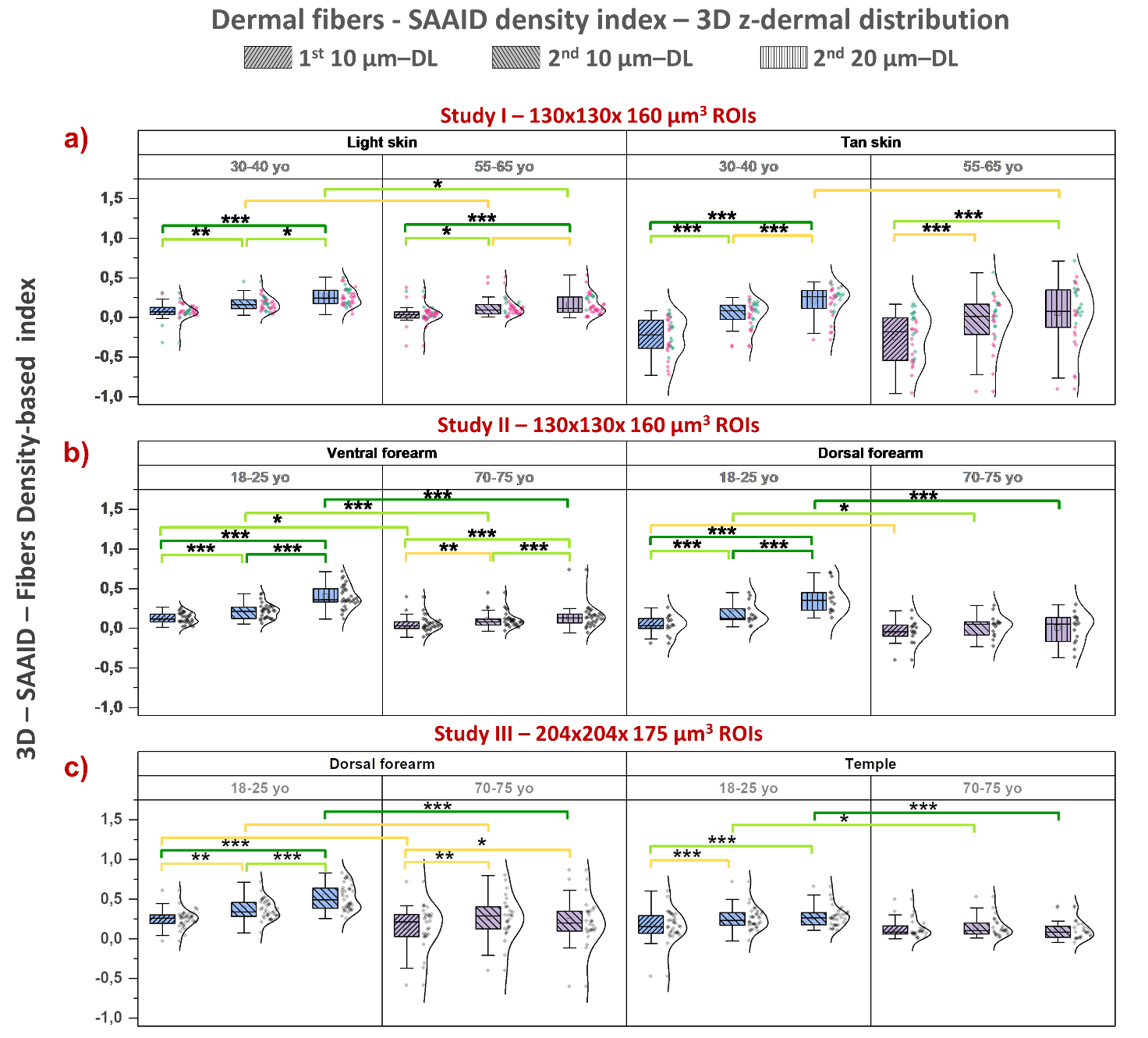


**Figure S 4: Multiphoton – Aging differences in the z-dermal distribution of the 3D – SAAID fibers density-based index.** Data from 3 clinical trials and within the 1^st^ 10 µm, 2^nd^ 10 µm and 2^nd^ 20 µm thickness normalized dermal sublayers (DL) that follow the DEJ shape: **a)** study I acquired with DermaInspect on ventral forearm (30-40 vs 55-65yo; “light” ITA vs “tan” ITA skin color groups); **b)** study II acquired with DermaInspect on ventral and dorsal forearms (18-25 vs 70-75yo); **c)** study III acquired with MPT*flex* on dorsal forearm and face temple area (18-25 vs 70-75yo). The data are expressed as box plots with fences, mean and median (—). All ROIs values and their distribution (dots (♦) and histogram) are shown on the right side of the boxplot. Overlapping values are highlighted in black (♦). In study I, the pink ♦ and green ♦ data points correspond to respectively women and men volunteers. All the statistical *p*-values and ES – effect sizes parameters are given in Table S4. Statistically significant *p*-values: *≤0.05; **≤0.01, *** ≤0.001. The colored brackets indicate the ES – effect size (dark green - very strong ]1.3 - Inf], light green - strong ]0.8 - 1.3] and yellow - moderate ]0.5 - 0.8]). Only *p*-values associated with moderate to very strong ES are shown.


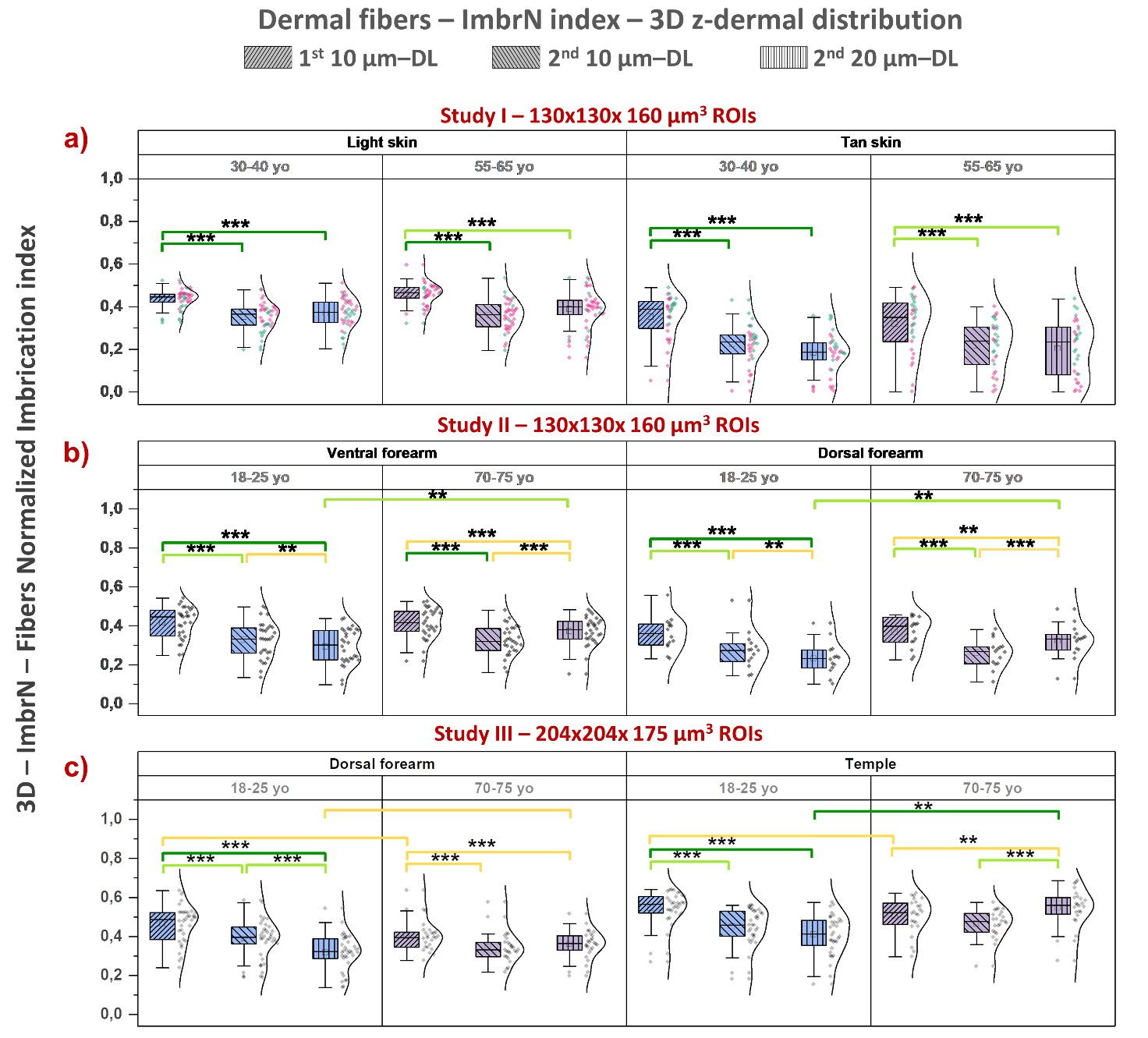


**Figure S 5: Multiphoton – Aging differences in the z-dermal distribution of 3D elastin / fibrillar collagen normalized imbrication index ImbrN.** Data from 3 clinical trials and within the 1^st^ 10 µm, 2^nd^ 10 µm and 2^nd^ 20 µm thickness normalized dermal sublayers (DL) that follow the DEJ shape: **a)** study I acquired with DermaInspect on ventral forearm (30-40 vs 55-65yo; “light” ITA vs “tan” ITA skin color groups); **b)** study II acquired with DermaInspect on ventral and dorsal forearms (18-25 vs 70-75yo); **c)** study III acquired with MPT*flex* on dorsal forearm and face temple area (18-25 vs 70-75yo). The data are expressed as box plots with fences, mean and median (—). All ROIs values and their distribution (dots (♦) and histogram) are shown on the right side of the boxplot. Overlapping values are highlighted in black (♦). In study I, the pink ♦ and green ♦ data points correspond to respectively women and men volunteers. All the statistical *p*-values and ES – effect sizes parameters are given in Table S4. Statistically significant *p*-values: *≤0.05; **≤0.01, *** ≤0.001. The colored brackets indicate the ES – effect size (dark green - very strong ]1.3 - Inf], light green - strong ]0.8 - 1.3] and yellow - moderate ]0.5 - 0.8]). Only *p*-values associated with moderate to very strong ES are shown.


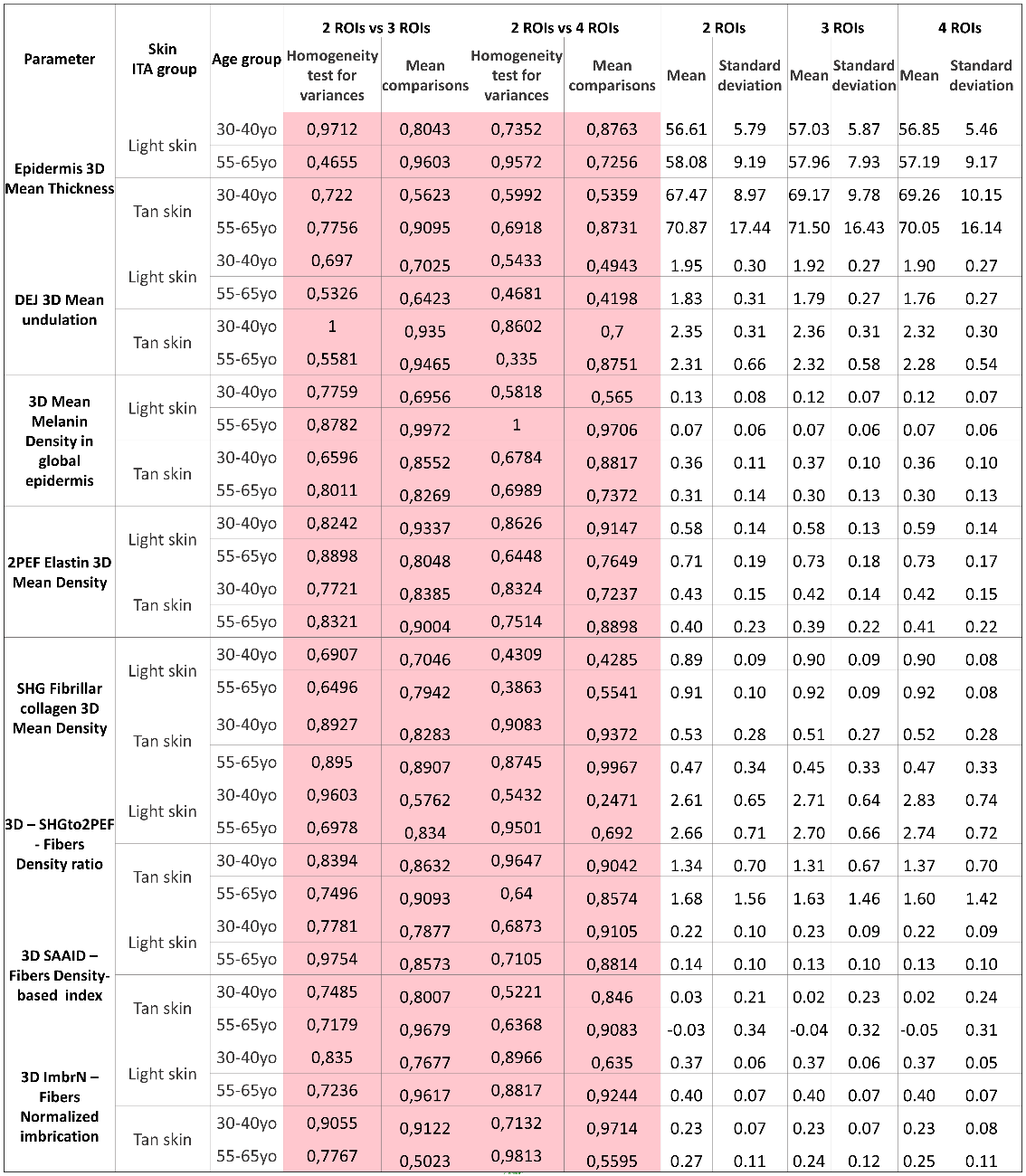


**Table S 1:** **Statistical analysis results (*p*-values) of the homogeneity tests for variances and means comparisons - Influence of the number of measurements (ROIs) on multiphoton 3D skin quantification parameters.** Study I acquired with DermaInspect on ventral forearm (30-40 vs 55-65yo; “light” ITA vs “tan” ITA skin color groups). Comparisons of data obtained with 2, 3 and 4 ROIs per volunteer. For each volunteer, an average value of the regions of interests was computed and the data for all volunteers are used to estimate the mean with its standard deviation for each subgroup. Interpretation *p*-values: statistically significant (very light green *p*≤0.05; light green *p*≤0.01; dark green *p*≤0.001), not significant (light orange 0.05< *p* ≤0.1 and light pink *p>*0.1).


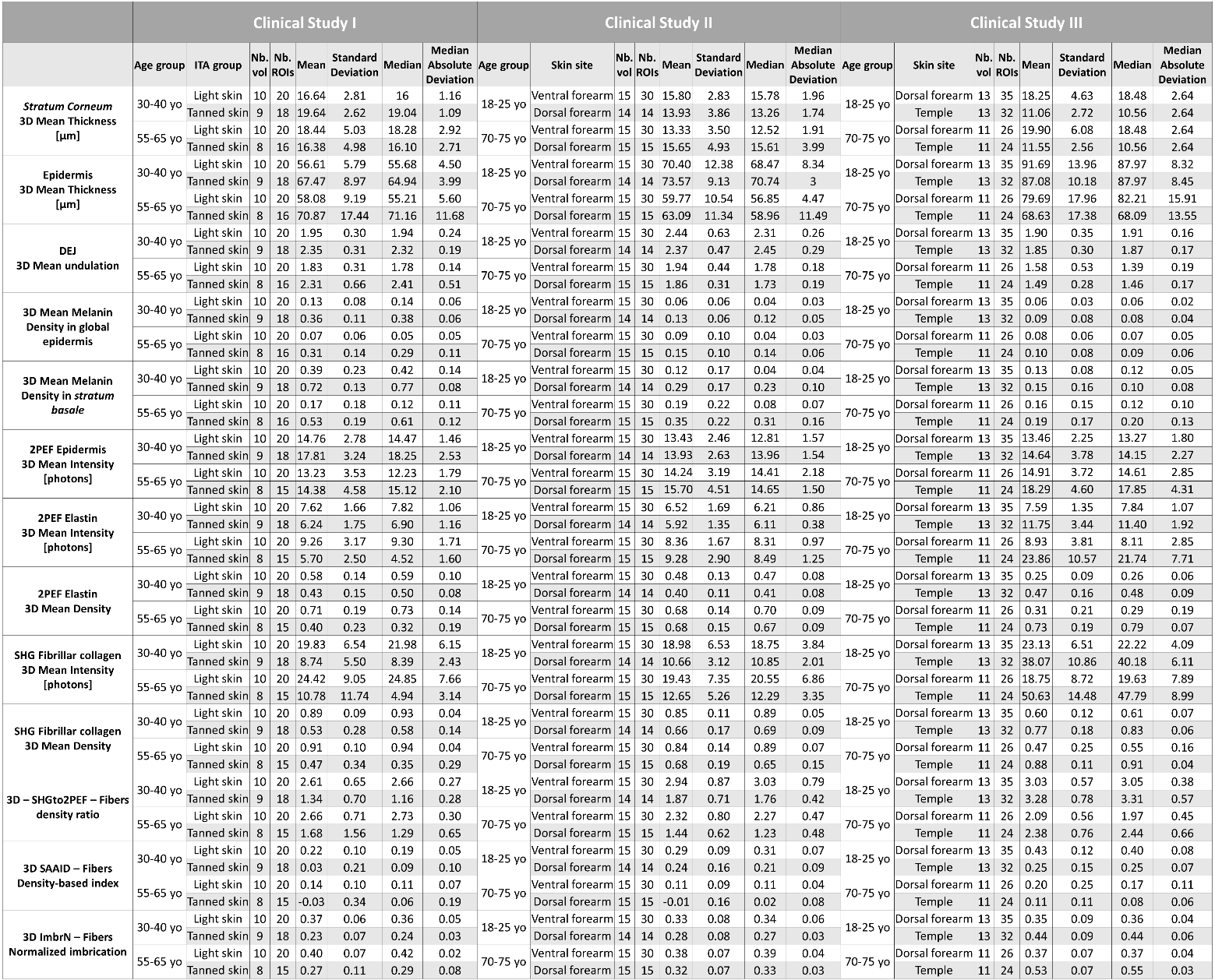


**Table S 2:** **Descriptive statistics of multiphoton quantification parameters for all clinical studies:** study I acquired with DermaInspect on ventral forearm (30-40 vs 55-65yo; “light” ITA vs “tan” ITA skin color groups); study II acquired with DermaInspect on ventral and dorsal forearms (18-25 vs 70-75yo); study III acquired with MPT*flex* on dorsal forearm and face temple area (18-25 vs 70-75yo. For each subgroup are given the total number of volunteers, of regions of interest (~2 per volunteer, not averaged), the mean with its standard deviation and the median with its median absolute deviation.


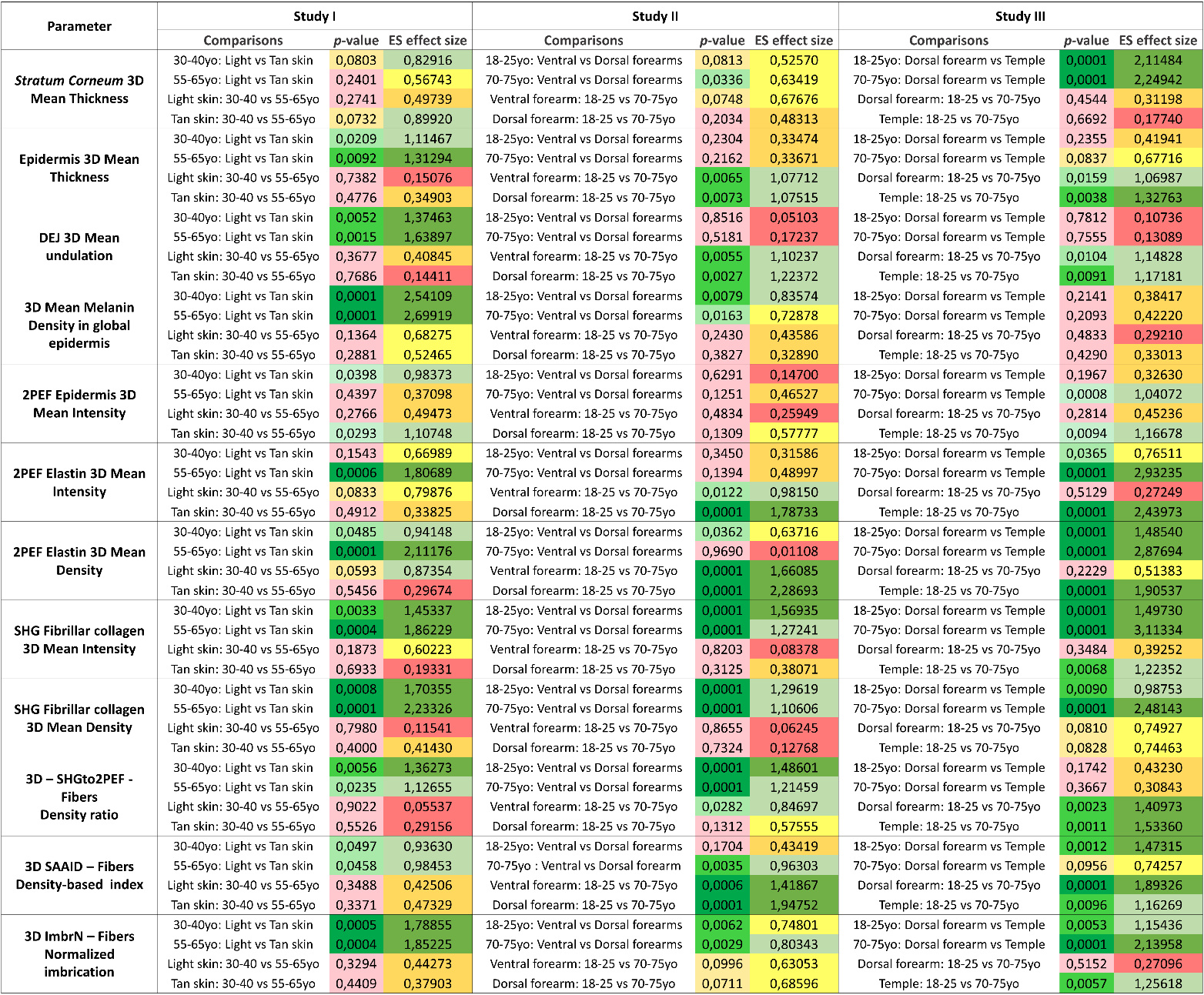


**Table S 3:** **Statistical analysis results (*p*-values and ES - effect sizes) of global multiphoton 3D quantification parameters of *in vivo* human skin.** Data from 3 clinical studies: study I acquired with DermaInspect on ventral forearm (30-40 vs 55-65yo; “light” ITA vs “tan” ITA skin color groups); study II acquired with DermaInspect on ventral and dorsal forearms (18-25 vs 70-75yo); study III acquired with MPT*flex* on dorsal forearm and face temple area (18-25 vs 70-75yo). For each parameter, the mean value of the individual ROIs data per volunteer and per experimental condition were used for statistical analysis. The number of volunteers is given in Table S2. The dermal parameters were estimated within 50 µm-thick dermal sublayer following the shape of the DEJ. Interpretation *p*-values: statistically significant (very light green *p*≤0.05; light green *p*≤0.01; dark green *p*≤0.001), not significant (light orange 0.05< *p* ≤0.1 and light pink *p>*0.1). Interpretation ES – effect sizes calibrated for multiphoton parameters (see Materials and methods): very strong ]1.3 - Inf] (dark green), strong ]0.8 - 1.3] (light green), moderate ]0.5 - 0.8] (yellow), weak ]0.3 - 0.5] (orange), very weak ]0 - 0.3] (pink) and no effect if ES=0.


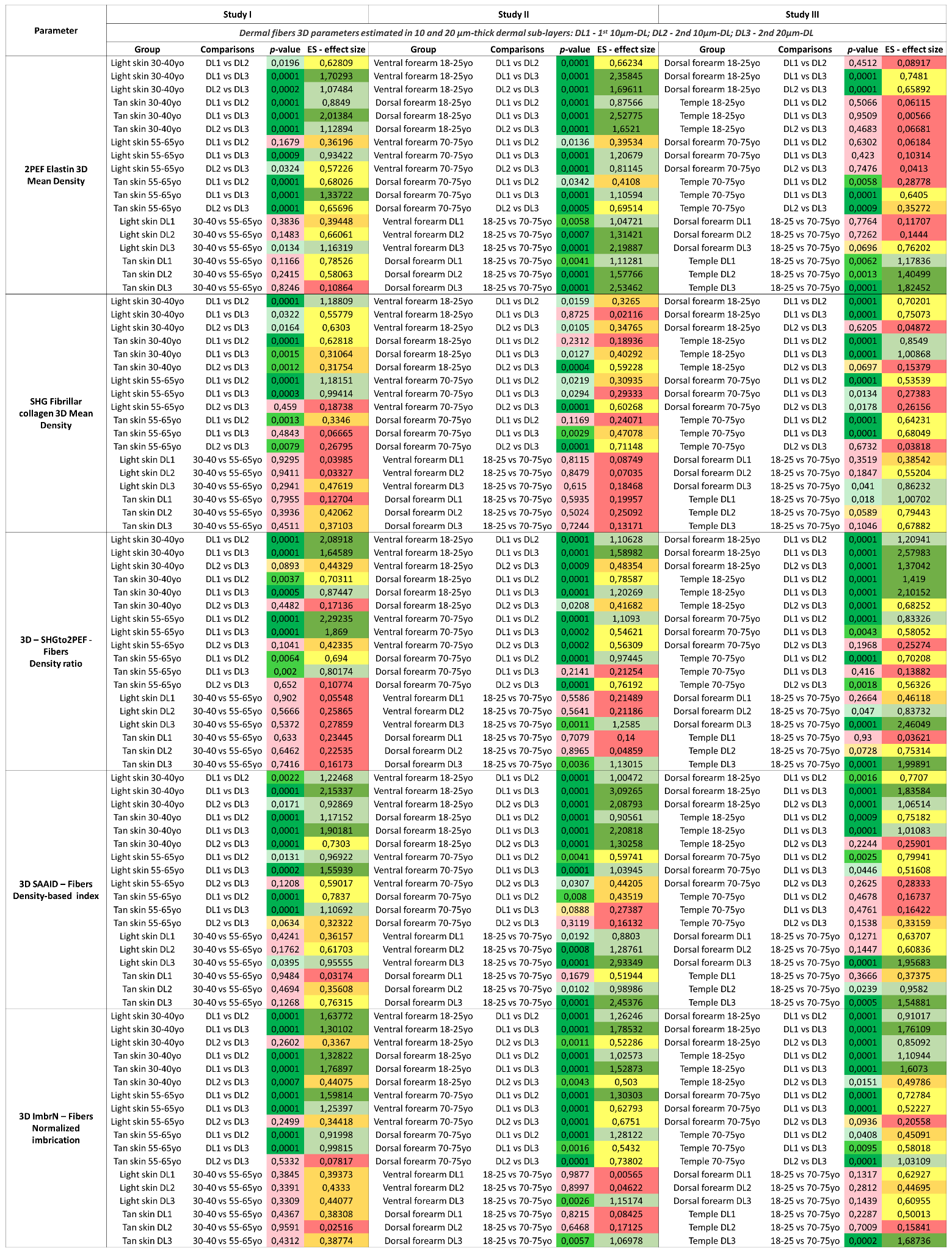


**Table S 4:** **Statistical analysis results (*p*-values and ES - effect sizes) of *in vivo* human skin multiphoton 3D quantification parameters characterizing elastin and fibrillar collagens within the z-dermal sublayers.** Data from 3 clinical studies: study I acquired with DermaInspect on ventral forearm (30-40 vs 55-65yo; “light” ITA vs “tan” ITA skin color groups); study II acquired with DermaInspect on ventral and dorsal forearms (18-25 vs 70-75yo); study III acquired with MPT*flex* on dorsal forearm and face temple area (18-25 vs 70-75yo). For each parameter, the mean value of the individual ROIs data per volunteer and per experimental condition were used for statistical analysis. The number of volunteers is given in Table S2. The dermal parameters were estimated within 10 and 20 µm-thick dermal sublayers following the shape of the DEJ. Interpretation *p*-values: statistically significant (very light green *p*≤0.05; light green *p*≤0.01; dark green *p*≤0.001), and not significant (light orange 0.05< *p* ≤0.1 and light pink *p>*0.1). Interpretation ES – effect sizes calibrated for multiphoton parameters (see Materials and methods): very strong ]1.3 - Inf] (dark green), strong ]0.8 - 1.3] (light green), moderate ]0.5 - 0.8] (yellow), weak ]0.3 - 0.5] (orange), very weak ]0 - 0.3] (pink) and no effect if ES=0.
